# Integrating *in vitro* experiments with *in silico* approaches for Glioblastoma invasion: the role of cell-to-cell adhesion heterogeneity

**DOI:** 10.1038/s41598-018-34521-5

**Published:** 2018-11-01

**Authors:** M.-E. Oraiopoulou, E. Tzamali, G. Tzedakis, E. Liapis, G. Zacharakis, A. Vakis, J. Papamatheakis, V. Sakkalis

**Affiliations:** 1 Department of Medicine, University of Crete, Heraklion Crete, Greece; 2 Computational Bio-Medicine Laboratory, Institute of Computer Science, Foundation for Research and Technology-Hellas, Heraklion Crete, Greece; 3 Institute of Electronic Structure and Laser, Foundation for Research and Technology-Hellas, Heraklion Crete, Greece; 4 Neurosurgery Clinic, University General Hospital of Heraklion, Crete, Greece; 5 Gene Expression Laboratory, Institute of Molecular Biology and Biotechnology, Foundation for Research and Technology-Hellas, Heraklion Crete, Greece; 6 Department of Biology, University of Crete, Heraklion Crete, Greece; 7 Present Address: Helmholtz Zentrum München, German Research Center for Environmental Health (GmbH), Neuherberg, Germany

## Abstract

Glioblastoma cells adopt migration strategies to invade into the brain parenchyma ranging from individual to collective mechanisms, whose role and dynamics are not yet fully understood. In this work, we explore Glioblastoma heterogeneity and recapitulate its invasive patterns both *in vitro*, by utilizing primary cells along with the U87MG cell line, and *in silico*, by adopting discrete, individual cell-based mathematics. Glioblastoma cells are cultured three-dimensionally in an ECM-like substrate. The primary Glioblastoma spheroids adopt a novel cohesive pattern, mimicking perivascular invasion in the brain, while the U87MG adopt a typical, starburst invasive pattern under the same experimental setup. Mathematically, we focus on the role of the intrinsic heterogeneity with respect to cell-to-cell adhesion. Our proposed mathematical approach mimics the invasive morphologies observed *in vitro* and predicts the dynamics of tumour expansion. The role of the proliferation and migration is also explored showing that their effect on tumour morphology is different per cell type. The proposed model suggests that allowing cell-to-cell adhesive heterogeneity within the tumour population is sufficient for variable invasive morphologies to emerge which remain originally undetectable by conventional imaging, indicating that exploration in pathological samples is needed to improve our understanding and reveal potential patient-specific therapeutic targets.

## Introduction

Glioblastoma (GB) is a very aggressive, highly infiltrative^1,2^ cancer of the Central Nervous System classified as grade IV glioma by the World Health Organization with multiple molecular subtypes^3^ and extensive intra-^4^ and inter-patient heterogeneity^5,6^. GB cells migrate into the neighbouring brain parenchyma and expand, characterizing GB as a diffusive rather than a focal disease^7^. It becomes evident that it is virtually impossible from a technical point of view to totally exempt the patient from the malignancy even in the case of gross resection^8^. As a result, tumour relapse may occur^9^ in the original or nearby brain regions^10^ from the invasive cells that are left over. On top of that, broad heterogeneity in GBs has been identified at the genotype, phenotype and molecular evolution level even within the same tumour, whereas spatially distinct tumour samples display different subtypes^11^. Inter-and intra-tumoural heterogeneity is a major biological property of GB tumours that reflects the continuous, spontaneous, and/or drug-driven evolution of cancer cells. GB is subject to clonal and epigenetic evolution, as well as microenvironmental forces that all together result in recurrence, therapy resistance and poor prognosis in spite of recent advances. The dynamic interplay of various sub-populations that coexist within a tumour further limits progress in implementing novel, effective treatment strategies. Although current treatment usually alleviates the symptoms, GB remains a clinical challenge exhibiting very poor prognosis with less than 10% of the patients having a 5-year survival rate^6^. Thus, it is evident why recapitulating the invasive morphology and dynamics is of great significance to eliminate clinical aggressiveness.

Invasion is a complex, multiscale phenomenon involving processes at different spatial and temporal scales. Migrating tumour cells can mechanistically move by different modes, ranging from single cell to collective locomotion, or even to whole-tissue expansion^12^. The molecular pathways during movement are complex and involve both energy utilization and response to stimuli, either chemical or mechanical or both. The invasive process necessitates both locomotion and proteolysis and involves both cell-to-matrix and cell-to-cell adhesion mechanisms. More specifically, it is believed that in multi-cellular invasion, transmembrane integrins are highly expressed at the “leading edge” tumour cell protrusions (pseudopodia), where they form focal contacts with the actin cytoskeleton. In addition, mechanical feedback through cell-to-cell junctions^13^ and/or cell adhesion proteins such as N- and E-cadherin (though the latter is believed to have limited expression in the brain) contribute to the collective migration of glioma cells by promoting direction sensing. Interestingly, differential expression of cadherins has been observed in GB samples, as well as disorganization and instability in cell-to-cell interactions^14–21^, supporting the presence of intratumoural heterogeneity with respect to cell-to-cell adhesion leaving open questions about its role in invasion.

A number of quantitative *in vitro* models have been developed over the past decades to study glioma invasion, most of which are based on the original trans-well or Boyden chamber assay systems^22–24^, where single cells invade from an upper chamber through an extracellular matrix (ECM)-like membrane or an ECM-coated filter to a lower chamber in response to chemoattractants. The latest trends in phenocopying GB in general and regarding invasion, mainly involve patient-derived cells -to individualize tumour properties^25,26^ and 3D *in vitro* experiments- to better mimic the parental tumour pathophysiology^27,28^. Tumour spheroids as a model system can be well characterized and have been shown to reproduce the spatial organization and micro-environmental factors of *in vivo* micro tumours, such as relevant gradients, establishment of cell-to-ECM adhesion and cell-to-cell interactions and deposition of ECM. Recent studies have shown that when glioma cells grow *in vitro* as multi-cellular spheroids, they are able to recapitulate invasive strategies observed *in vivo* including the collective behaviour^29,30^.

Given the complexity, an increase in mathematical modelling research has been observed the last decades in an attempt to systematically integrate information from multiple biological experiments and to provide better understanding of the potential underlying mechanisms involved and their impact on GB motility, dissemination, invasion and morphology. The mathematical approaches mainly lie into two broad categories of discrete and continuous mathematics. *Continuous mathematical models* focus on the averaged behaviour of tumour cells and describe tumour and microenvironment at tissue level. On the other hand, *individual-cell-based models* using discrete and hybrid discrete-continuous mathematics address the behaviour of each cancer cell individually, bridging the scaling gap between cells and tissues. These models have been proven extremely powerful systems as they are capable of producing a variety of complex behaviours from simple rules. Individual-cell-based models are in general more suitable to describe *in vitro* experiments, genotype-phenotype relations, local interactions of heterogeneous populations and migration mechanisms. A comprehensive overview of the mathematical models developed for GB progression and therapy response from the clinical perspective and personalized medicine are summarized in^31^. In addition, a thorough review summarizing major studies related to GB invasion can be found in Alfonso *et al*.^32^. Among these studies, the particular importance of the microenvironment and the central role of cell-to-cell and cell-to-ECM interactions on the evolution of invasion are extensively explored, as well as the mechanisms of phenotypic plasticity and adaptation. Nevertheless, most models focus on single-cell migration phenomena. Furthermore, the role of intra- and inter-tumoral heterogeneity and particularly with respect to cell-to-cell adhesion properties, is less studied. Anderson^33^ accommodates in his model phenotypes with different adhesion properties, however these properties are subject to mutations and thus, vary through time. In that approach, additional properties of cancer cells, including their proliferation and migration rates that can supplant the role of heterogeneous cell-to-cell adhesion interactions, are also involved. Domschke *et al*.^34^ studied the role of cell adhesion variability on the invasive pattern formation. In their model, variability is taken into account again in a time-dependent manner, where cancer cells sequentially mutate into more aggressive phenotypes with respect to cell-to-cell and cell-to-matrix adhesion properties. Furthermore, the local interplay of neighbouring cells is not considered. Reher *et al*.^19^ systematically explored the effect of both intrinsic and extrinsic cues of adhesion heterogeneity yet, specifically on tumour cell dissemination. Overall, none of these studies focuses on the intrinsic heterogeneity with respect to the interplay of co-existing phenotypes with different cell-to-cell adhesion properties and its impact on alternative invasion patterns.

In this work, we study the invasive potential of GB cells under a set of basic experimental parameters, by means of forcing both U87MG cells and an in-house-established primary GB cell line to form 3D cell cultures at an ECM-like substrate. Our biological experimental results consistently show that the two types of tumour spheroids display different invasive patterns, suggesting that different mechanisms of cell motility are adopted by the two cell lines. An individual-cell-based computational model is adopted, accounting for heterogeneity in cell-to-cell adhesion properties of the cells to predict the variety of the invasive morphologies and kinetics observed. Improving our understanding of the underlying mechanisms, which drive and/or regulate the different invasion patterns observed among GB subtypes will offer opportunities for alternative and GB type-specific drug targets to prevent post-operative tumour relapse. Furthermore, predicting the various invasive morphologies will potentially help to better assess the extension of invasion, which remains undetectable by conventional imaging modalities.

## Results

### *In vitro* experiments

The invasion of primary and U87MG GB spheroids was studied in this work. Doubling time estimation experiments (as described in the Supplementary text) showed that both cell types are highly proliferative with cell population mean doubling times of 30.8 h and 25.4 h for the U87MG and the primary cell line, respectively. In the 3D invasion assay, cell migration was fully ECM-dependent, since no invasion was observed in its absence. Spheroids were monitored over a total period of 12 days and the invasive patterns formed were consistently observed in all the experiments per cell type.

### Invasive pattern of U87MG cells over time

**Figure 1:**
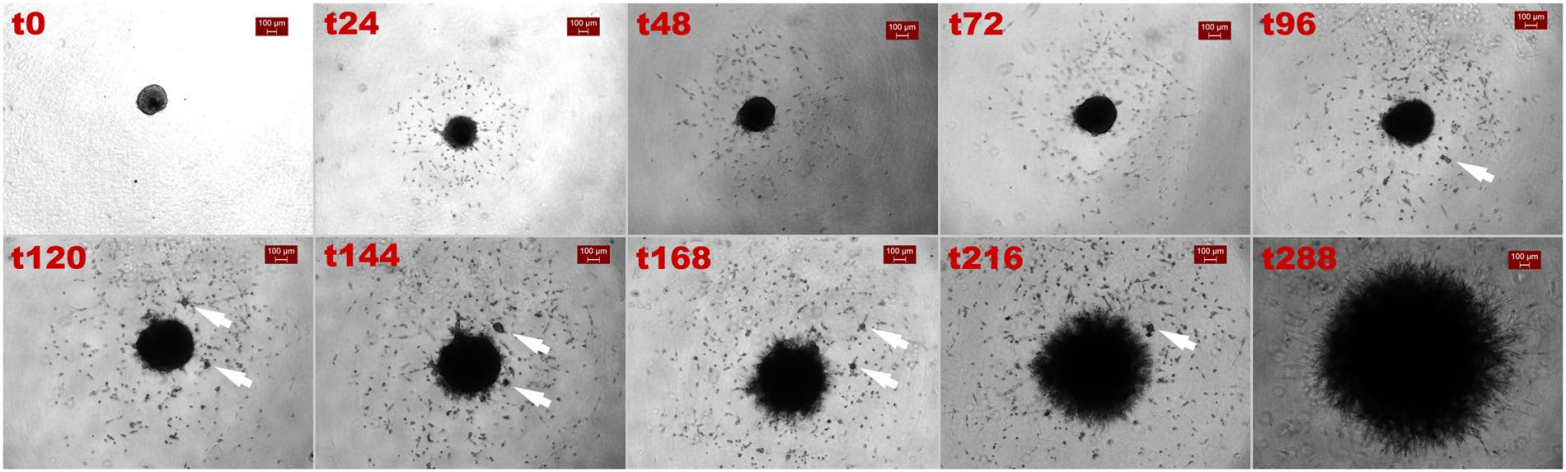
Invasion of the U87MG spheroids over time. Brightfield images at a 4x magnification and scalebar is set at 100 μm. White arrows indicate cell aggregates.

Fig. 1 presents consecutive brightfield images of a representative U87MG spheroid undergoing invasion within a 24-hour time interval (excluding the last two images, t216 and t288). As shown in Fig. 1, U87MG cells exhibited an immediate invasive phenotype within the first 24 h after seeding. They extended symmetrically from the core maternal spheroid towards the periphery, within the ECM-like substrate, following a non-cohesive migration pattern. In accordance with relevant studies^24,28,35^, random prolonged cellular protrusions were also observed; yet no noticeable cell path track in the ECM was detected in the brightfield images. This type of outgrowth behaviour continues until approximately 72 h with slight variation. After 96 h, the most distant cells had reached the boundaries of the well. In line with previous reports^35^, at this time, satellite cell clusters were also starting to form, and invasion adopted a more complex dynamic behaviour. Interestingly, after 288 h of allowed invasive condition with no nutritional exhaustion, the surrounding aggregates seemed to deform, whilst the maternal spheroid, that had remarkably grown, had no more defined borders, while all peripheral cells were prolonged.

### Invasive pattern of primary cells over time

**Figure 2:**
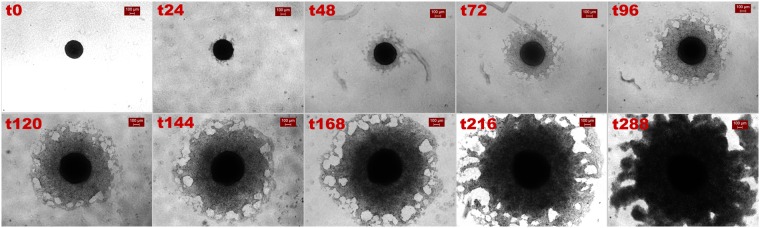
Invasion of the primary GB spheroids over time. Brightfield images at a 4x magnification and scalebar is set at 100 μm.

Primary GB spheroids adopted an apparently alternative, cohesive invasive morphology with boundary instabilities, not reported before in relevant studies^12,29,30,36,37^. Fig. 2 illustrates the evolution of the invasion pattern of a representative primary spheroid. The same invasive pattern was consistently observed in all primary GB spheroids of the same patient that we tested. Initially, few invasive cells seem to asymmetrically exit away from the maternal core spheroid towards the periphery. At intermediate time points, the invading cells appear to collectively form a cohesive, sheet-like structure (as described in^12^). Finally, in the following time points, until 288 h, the invasive pattern appears unaltered, but still enhanced.

### Growth dynamics of tumour spheroids

**Figure 3:**
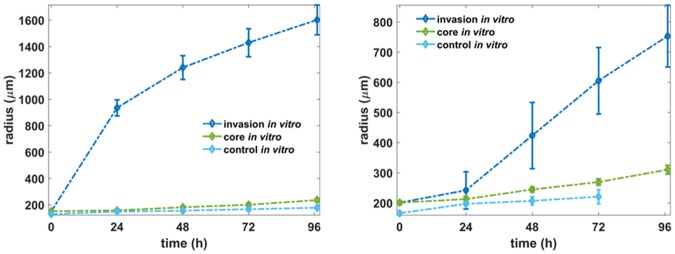
Time evolution of tumour core and invasive radii for the U87MG (left) and the primary (right) spheroids with and without the invasive condition. The radii from twenty spheroids per timepoint were analysed using regression analysis. The error bars denote the standard deviation.

Fig. 3 shows the temporal evolution of the average values of the core and invasive radii from all the experiments for both the U87MG and the primary spheroids, based on the segmented brightfield images. The time evolution of the negative control experiments is also depicted. Considering that after 96 h, the most distant invasive cells of the U87MG spheroids reach the boundaries of the well, we focus on this time period for both cell lines.

The invasive radius of the U87MG spheroids showed a rapid expansion the first 24 h that slowed down at later times. The opposite behaviour is observed for the core radius. The invasive radius of the primary spheroids displayed a slow expansion during the first 24 h that was followed by a faster linear expansion. Various mechanisms can affect the motility of the GB cells *in vitro*, including stress (as the cells are transferred to an invasion matrix^28^) and ECM production by the tumour cells^27^, which dynamically alter their kinetics. Although interesting, the exact underlying molecular mechanisms involved in motility regulation are beyond the scope of the present study. In both the U87MG and the primary spheroids, the core radius evolves slower than the invasive radius. After the first 24 h, the mean expansion speed of both cell lines is similar and equals to 7.1 and 9 μm/h for the primary and the U87MG cells, respectively. Nevertheless, in the first 24 h the expansion speed is considerably different and estimated equal to 1.7 and 32.7 μm/h for the primary and the U87MG cells, respectively.

### *In silico* experiments

The *in silico* tumour was initialized to a size close to the initial tumour size of the biological experiment and grew for 9 days unless a cell reached the edge of the computational domain within a proximity of 5 cells. Thus, a disc of size approximately 140 μm in radius for the U87MG and of 200 μm for the primary cells located in the centre of the computational domain, was initially assumed completely filled with cancer cells. The simulations were repeated 50 times for each cell line. Variation in the computational results derived from the randomness in the cellular movement and the arbitrary initialization of cellular phenotypes and cell age. To describe the different invasion patterns observed, we assumed that tumours are composed of phenotypes with different adhesive properties. To quantitatively assess the morphology and growth of the simulated tumours, we used the metrics presented in the Supplementary Text. Specifically, we focus on the temporal evolution of the core and the invasive radii, as well as the local compactness and local sparseness of the tumour. Unless otherwise stated, the set of all the parameters used in our simulations are depicted in Supplementary Text: Tables 1 and 2.

### Different mixture of phenotypes produces different morphologies

**Figure 4:**
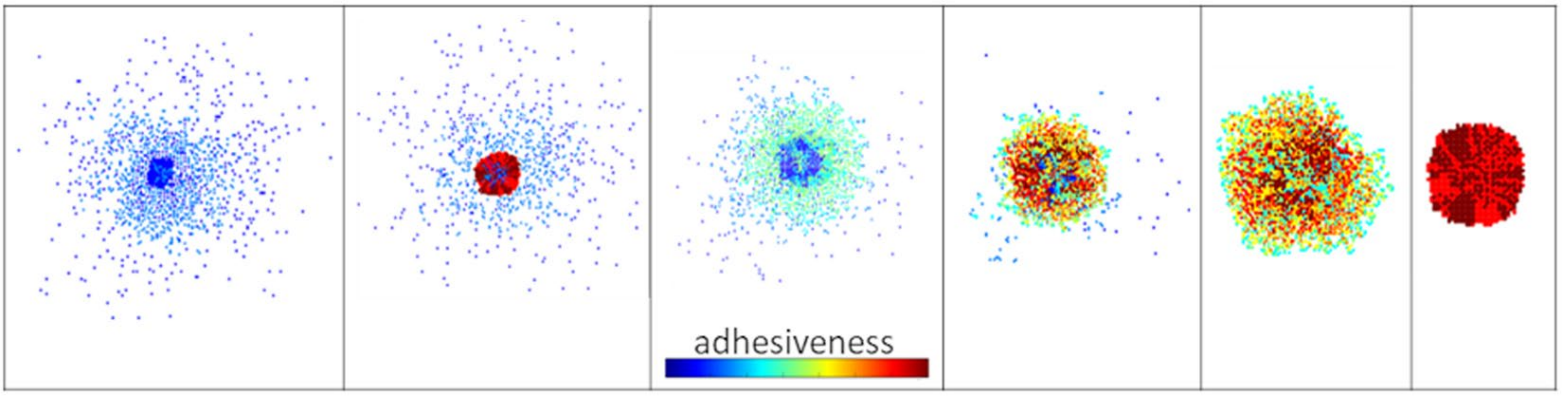
Snapshots of various morphologies emerging by combining phenotypes with different cell-to-cell adhesion properties. Cell-to-cell adhesiveness ranges from 0 (blue) to 7 (red) colour indicating low to strongly adhesive phenotypes, respectively. All snapshots are captured at the end of the simulation process, which corresponds to 112, 160, 144, 184, 216 and 216 h respectively, from left to right.

A spectrum of different morphologies arises when phenotypes of various cell-to-cell adhesion properties are combined. These morphologies vary from highly compact, where invasion is hardly observed, to cohesive patterns and even to non-cohesive migration patterns, under the same microenvironmental conditions. As expected, highly adhesive phenotypes strongly attract and are attracted by many other cells, thus forming dense and symmetric patterns with limited motility and reduced invasive radius. On the other hand, phenotypes with loose cell-to-cell interactions adopt non-cohesive migration strategies and travel unbiased further away from the maternal spheroid, showing decreased compactness and increased invasive expansion and sparseness. Interestingly, the interplay of these phenotypes can produce a variety of different dynamics for the expansion of the core and invasion radii, as well as a variety of morphologies with different overall compactness and sparseness, as indicatively shown in Fig. 4 and Supplementary Text: Fig. I. In this set, all the experiments were performed with fixed proliferation and diffusion rates equal to 31 h and $\,5\cdot {10}^{-9}c{m}^{2}/s$, respectively.

### Phenotypes of low and high adhesiveness resemble the invasive pattern of the U87MG spheroids

**Figure 5:**
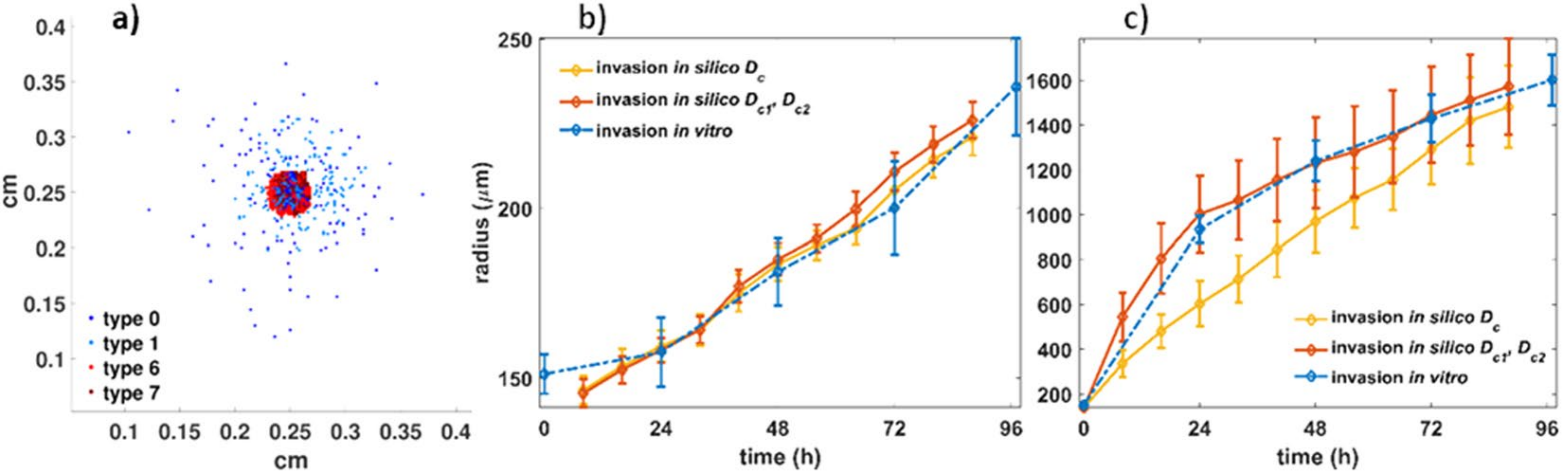
*In silico* predictions of the U87MG cell type: (**a**) Snapshot of the simulated U87MG spheroid at 96 h (left), (**b**) the temporal evolution of the core radius, and (**c**) the invasive radius for both the *in vitro* and *in silico* experiments over time.

We observed that in order to describe the U87MG cell line, low adhesive phenotypes and highly adhesive phenotypes should be considered. The latter are necessary to describe the maternal immotile core, while the former represent the highly migrating invasive cells. Fig. 5a shows the simulated results of the U87MG invasive spheroids at 96 h. Few phenotypes of low adhesiveness can also be observed trapped within the core due to spatial competition. The simulated evolution of the U87MG invasive spheroid can be seen in the Supplementary video SV1 and the Supplementary Text: Fig. J. The proliferation time was set equal to 31 h and the diffusion coefficient was set equal to *D*_*c*_ = 5·10^−9^ *cm*^2^/*s*.

### Phenotypes of middle and high adhesiveness resemble the invasive pattern of the primary spheroids

**Figure 6:**
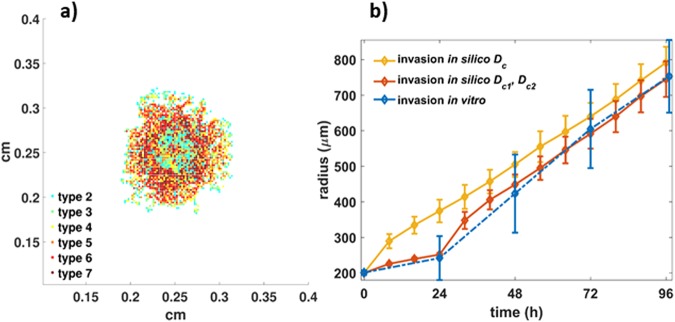
*In silico* predictions of the primary cell type: (**a**) snapshot of the simulated primary spheroid at 96 h (left), (**b**) the temporal evolution of the invasive radius of both the *in vitro* and *in silico* experiments.

To recapitulate the cohesive primary cell line morphology, phenotypes with middle to strong cell-to-cell adhesive interactions were assumed. Low adhesive phenotypes were excluded from this experiment. We should note that alternative combinations of phenotypes may possibly produce similar results, as for example using only the middle adhesive phenotypes. However, as can be seen in Supplementary Text: Fig. I, in that case the tumour compactness initially decreases and only after a period of time increases forming a compact core. On the contrary, including phenotypes with high adhesion, an almost immediate increase in tumour compactness was observed, better resembling the core expansion of the *in vitro* experiments. The diffusion coefficient was set to ${D}_{c}=2\cdot {10}^{-8}c{m}^{2}/s$ and the proliferation time was set to $25\,h$, in accordance to the doubling time estimate. A snapshot at 152 h of the tumour evolution is illustrated in Fig. 6a (see also Supplementary video SV2 and the Supplementary Text: Fig. K). As can be seen in Fig. 6b, apart from the trapped cells in the core, we can observe that relatively low adhesive phenotypes (types 2, 3, 4) tend to appear in the tips of the tumour sprouts, while phenotypes with relatively stronger cell-to-cell adhesive interactions (types 5, 6 and 7) are more likely to be found closer to the tumour core. Interestingly, all phenotypes coexist within the tumour, increasing their populations as tumour evolves, with the phenotypes of types 4 and 5 to be systematically present at higher frequencies (see also Supplementary Text: Fig. L). Moving towards the centre, the tumour becomes denser and after approximately 150 h necrotic cells start to appear. It is noteworthy that as time passes, new gaps are formed, while the gaps already formed between the sprouts gradually close without trapping any of the highly adhesive phenotypes.

### Temporal evolution predictions of spheroid expansion

Note that, *in silico*, for the primary cell type, we cannot distinguish the core from the invasive area based on the distribution of phenotypes, as we can do for the U87MG spheroids due to their mixed spatial distribution. Thus, for the primary spheroids, we focus only on the temporal evolution of the invasive radius. Furthermore, in order to better approximate the different kinetics observed before and after the first 24 h, we assumed two distinct phases in tumour expansion governed by different motility rates in addition to the single motility rates. Specifically for the U87MG spheroids, we set the diffusion coefficient in the time period [0, 24]h equal to ${D}_{c1}=1.5\cdot {10}^{-8}\,c{m}^{2}/s$ and for the rest period equal to ${D}_{c2}=3\cdot {10}^{-9}\,c{m}^{2}/s$. On the other hand, for the primary GB cell line, we assumed ${D}_{c1}=4\cdot {10}^{-10}c{m}^{2}/s\,\,$and $\,{D}_{c2}=4\cdot {10}^{-8}c{m}^{2}/s$. The temporal evolution of the expansion for both the U87MG and the primary spheroids is shown in Figs 5 and 6, respectively. The relative *in vitro* observations are also shown for direct comparison. By allowing different motility rates at the different growth phases the *in vitro* and the *in silico* curves converge for both cell types.

### The role of proliferation and motility rates

Variation in proliferation time and diffusion coefficient affects overall tumour growth and morphology (Table 1 and Supplementary Text: Figs M–X). Specifically, for the U87MG simulations, increased proliferation rate substantially affects the cell population, increases the expansion rate of the core and also affects the expansion rate of the invasive radius. On the other hand, increased motility rate considerably increases the invasive radius, but only slightly affects the expansion of the core and the cell population. Note that counterintuitively, increasing the proliferation of the U87MG cell type results in decrease of the overall compactness and increase of the sparseness after a time period. Due to the significantly less space competition, the outgrowth of the invasive cells is favoured relative to the growth of the core cells.

**Table 1:** The impact of proliferation, migration and phenotypic switch on tumour evolution.

Cell type	Rates	Cell population	Invasive radius	Compactness	Sparseness	Dominance of phenotypes
U87MG	Proliferation ↑	↑	↑	↓	↑	Type 6,7 ↓ Type 0,1 ↑
	Motility ↑	↑	↑	↓	↑	Type 6,7 ↓ Type 0,1 ↑
	Phenotypic switch ↑	↔	↔	↑	↔	↔
Primary	Proliferation ↑	↑	↑	↑	↓	Type 6,7 ↓ Type 2,3,4 ↑
	Motility ↑	↑	↑	↓	↔	Type 2,7 ↓ Type 4,5,6↑
	Phenotypic switch ↑	↓	↓	↑	↔	↔

Notably, for the primary spheroids, proliferation strongly affects the expansion of the invasive radius, as well as the cell population. Increased proliferation also results in more smooth and round tumours (Supplementary Text: Fig. P) increasing tumour compactness and reducing sparseness. Similarly, increasing the motility rate of the primary cells results in increase of both the invasive radius and the cell population as it allows more free space for cell growth and motility. Increase in motility, rate considerably decreases the compactness of the spheroid and increases sparseness.

Interestingly, variations in proliferation and motility rates also alter the relative frequency of phenotypes, as summarized in Table 1 and in the Supplementary Text: Figs N and O for the U87MG and Figs R and W for the primary GB spheroids, respectively. Overall, for the primary GB spheroids, we observed that by either increasing the motility of the cells or decreasing their proliferation, less compact tumours are formed, allowing more free space for the middle adhesive phenotypes to relatively increase their population. We should note however, that by selectively inhibiting the proliferation of the middle adhesive phenotypes, the highly adhesive phenotypes dominate in the population, forming fully compact tumours (Supplementary Text: Fig. T).

### Phenotypic switch

An intrinsic state transition probability of tumour cells was introduced to allow them to stochastically change phenotype during mitosis with probability equal to 0.5. For the simulations regarding the U87MG cells, we assume four possible phenotypes with adhesive values 0, 1, 6 and 7 (low and highly adhesive). All transitions among these phenotypes are possible and are equally likely. For the simulations of the primary cells, we assume six possible phenotypes (middle and highly adhesive) with adhesive values 2, 3, 4, 5, 6 and 7. Again, each phenotype has an equal probability of being selected. The new phenotype was randomly chosen and applied to both daughter cells. The result of this phenotypic switch was that now the self-organization of cells reflected in the diverse frequency of each phenotype as tumour evolves is not evident and all phenotypes involved have equal representation in the population. Regarding the U87MG cell type in particular, although slight changes were observed in the tumour expansion (Supplementary Text: Fig. Y), interestingly, cell aggregates peripherally to the maternal spheroid (Supplementary Text: Fig. Z), similar to those appearing at the later stages of U87MG spheroids invasion (Fig. 1). Interestingly, regarding the primary GB spheroids, we observed that an equal contribution of all phenotypes in the tumour composition introduces an eventual decrease of the overall cell population and tumour expansion and prompts the formation of a denser tumour (Supplementary Text: Figs BB and CC), similar to the morphology observed after 120 h in the respective biological experiment (Fig. 2). Thus, allowing random phenotype transition in both cases could possibly predict the morphologies observed at later time points, although alternative mechanisms triggered by the evolving tumour microenvironment and not necessary requiring mitosis could account for these morphologies too. Even more, a microenvironmental regulated phenotypic switch could also be a potential mechanism explaining the evolution of the invasion pattern.

## Discussion

In this work, we explored the invasive potential of GB cells using a rather simple, but yet realistic, set of experimental parameters. We utilized patient-derived cancer cells of a GB patient along with the established and commonly used U87MG cell line. GB cells are cultured in 3D in an ECM-like substrate. Our biological experiments show that the two types of tumour spheroids display considerably distinct invasive patterns suggesting different mechanisms of cell migration. In an attempt to explore possible mechanisms involved, an individual cell-based mathematical approach was adopted to indicate the potential role of the intrinsic heterogeneity with respect to cell-to-cell adhesion on tumour morphology and growth dynamics.

We implemented the 3D tumour spheroid invasion assay^28,38^ in order to investigate the initial steps of invasion from spheroids formed using single cell suspensions. The main advantage of this assay as compared to standard trans-well assays is that it can recapitulate the basic 3D structure of tumours and replicate features of collective cell invasion observed *in vivo*. In addition, this is a simple, quick and standardized assay that enables analysis of invasion with high reproducibility in a 96-well plate format. However, we should note that monitoring of invasion in the existing 3D spheroid invasion assays relies on brightfield imaging of the spheroid from the bottom of 96-well U-plates, which confines microscopic analysis of 3D spheroids to a 2D plane leading to exclusion of cell clusters invading in the depth dimension.

Based on the *in vitro* invasive protocol followed here, the two GB cell lines used, exhibited a markedly different invasive pattern. In consistence with other studies^24,28,36,38–41^, U87MG cells appeared to colonize the ECM via a process indicating non-cohesive, starburst migration. On the other hand, the GB primary spheroids kept expanding to massively conquer the surrounding regions rather than individually migrating potentially governed by homotypic attraction^42^. A unique, collective invasive pattern with morphological instabilities of cohesive protrusions near the boundary resembling perivascular invasion in the brain^29^ was observed. It is well recognized that exploring the physiological and molecular patterns of these cells might enable the design of novel therapeutics targeting the recurrence process. The ability to early detect the phenotypic composition of an evolving tumour is undoubtedly of significant prognostic value.

In order to further investigate potential intrinsic mechanisms involved in the invasion patterns observed, an individual-cell-based computational model accounting for intratumoural heterogeneity was developed. More specifically, different cell-to-cell adhesive properties adopted by the GB cells were assumed, although additional or even alternative mechanisms could also play a role in the observed tumour behaviour. Reher *et al*.^19^ have extensively studied mathematically the role of cell adhesion heterogeneity specifically on cell dissemination, opening the question of whether this heterogeneity is present in gliomas and how it affects the migration mechanisms and tumour morphology. In support to our work, recent studies^14–21^ have shown differential expression of cadherins, as well as observable disorganization and instability in cell-to-cell interactions within various GB cell lines. Primary cells most usually overexpress cell adhesion molecules, such as integrins or cadherins, whilst common/established cell lines do not^19,43–45^. Furthermore, complementing cell-to-cell, cell-to-ECM interactions were also shown computationally to play an important role in tumour invasion, with cell-to-cell interactions affecting predominantly the invasion pattern and cell-to-ECM influencing the invasion speed^33,46^. A variety of mathematical models have been developed to describe the emergence of invasion in cancer cells and GB specifically, as summarized in Alfonso *et al*.^32^. Yet, to the best of our knowledge, none of these studies focuses on the formation of invasive patterns, by taking into account the interplay of co-existing phenotypes with different cell-to-cell adhesion properties on tumour evolution and morphology. In this work, tumour expansion and morphology were explored and compared with the *in vitro* experimental data. Tumour expansion was quantitatively evaluated based on the temporal growth of the tumour spheroid core and the invasive radii. Furthermore, additional metrics including the locally derived sparseness and compactness were used to describe the morphologies. In general, tumour expansion is attributed to both the proliferative and migratory capacity of tumour cells. Thus, their role on tumour morphology and evolution was also investigated under the proposed framework.

Interestingly, we showed that by selecting (during model initialization) phenotypes with different cell-to-cell adhesion preference to coexist within the tumour is sufficient to resemble the distinct invasion patterns and the expansion rates we observed *in vitro* between the primary and the U87MG cells. We also observed that variation in proliferation time and diffusion coefficient affects overall the tumour compactness, sparseness, as well as the tumour expansion rates and changes the relative frequency of phenotypes according to cell type, indicating potential mechanisms that could alter tumour evolution and inhibit invasion. Forcing a strong dependence between adhesiveness and proliferation to mimic a potential “go-or-grow” mechanism (Supplementary Text: Figs M and N), we observed that although for the U87MG cells such hypothesis could possibly apply, proliferation plays a more complex and important role for the primary cells under the specific modelling assumptions. Interestingly, we also observed that by allowing cells to randomly switch phenotypes throughout tumour evolution, the self-organization of cells, reflected in the diverse frequency of each phenotype, was lost and all phenotypes involved have equal representation in the population with an impact on the evolution of the primary cell type. More specifically, in the primary tumours, we observed that by disabling the phenotypic switch, both the total tumour population and expansion increased, indicating that random phenotypic switch with respect to cell-to-cell adhesion does not favour tumour evolution.

It has to be noted that though the main aim of this work was to describe the different invasive morphologies experimentally observed, hypotheses of environmentally-triggered motility, such as the “go-or-grow”^47^ and/or hypoxia-driven migration^48,49^ regarding the proliferation to migration and/or adaptation to cell death switch, would be interesting to be included in our future work in order to explore their role in tumour morphology and dynamics. Additionally, it would be also interesting to extend our proposed mathematical model in 3D and explore whether and to what extent the observed morphologies are affected; although the work of Anderson^50^ has shown very similar invasive patterns between the 2D and 3D implementation of his model. On top of that, the effect of a more realistic description of the motility in a lattice-free framework that does not limit the possible directions of cell movement^51^ would be also of interest.

GB cells have been shown to exhibit a different invasive phenotype among different ECM components^52,53^, mainly regarding collagen type^54,55^ and rigidity/stiffness^56–58^. In addition, GB spheroids are also able to self-produce ECM^59^, while ECM deposition dynamically changes over time, a fact that should also be taken into account in our future investigations. Use of time-lapse cell migration monitoring will be of importance to verify the direction and velocity of cell movement, as well as the sprouting development. In future studies, more advanced imaging modalities should also be employed, such as light-sheet fluorescence microscopy (LSFM) or multispectral optoacoustic imaging (MSOT), which offer superior resolution at the sub-cellular level^60^. In combination with optical reporters of cell physiology, i.e. apoptosis, cell junctions, cell division, neural markers, etc., it will be of great benefit to further dissect the GB invasion properties and even better approximate the cellularity within a given tumour volume. Another technique that could be beneficial as a measure of compactness of the spheroids could be the immunohistopathological examination of permanently fixed spheroids, where specific markers of cellularity are available. On top of that, more advanced hybrid spheroid 3D invasion assays, such as co-cultures with organotypic brain slices^61^ and microfluidic platforms^62,63^ are still under development and could be used to better recapitulate *in vivo* conditions accounting for interactions among different cells, shear forces and vasculature.

In-depth understanding of different invasion patterns among GB subtypes^3^ and its potential mechanisms that might drive/regulate the observed heterogeneity will offer opportunities for alternative drug targets to prevent GB relapsing post-operatively and improve our understanding of the extension of invasion, which still remains undetectable by conventional imaging modalities. Overall, we propose that by advancing our mathematical approaches and taking advantage of *in vitro* experimental approaches, which enable tight control of experimental parameters and high reproducibility, it may be possible to eventually verify the precise set of their computational counterparts needed towards a systematic *in silico* mapping of GB invasion and progression.

## Methods

### Biological sample collection

**Figure 7:**
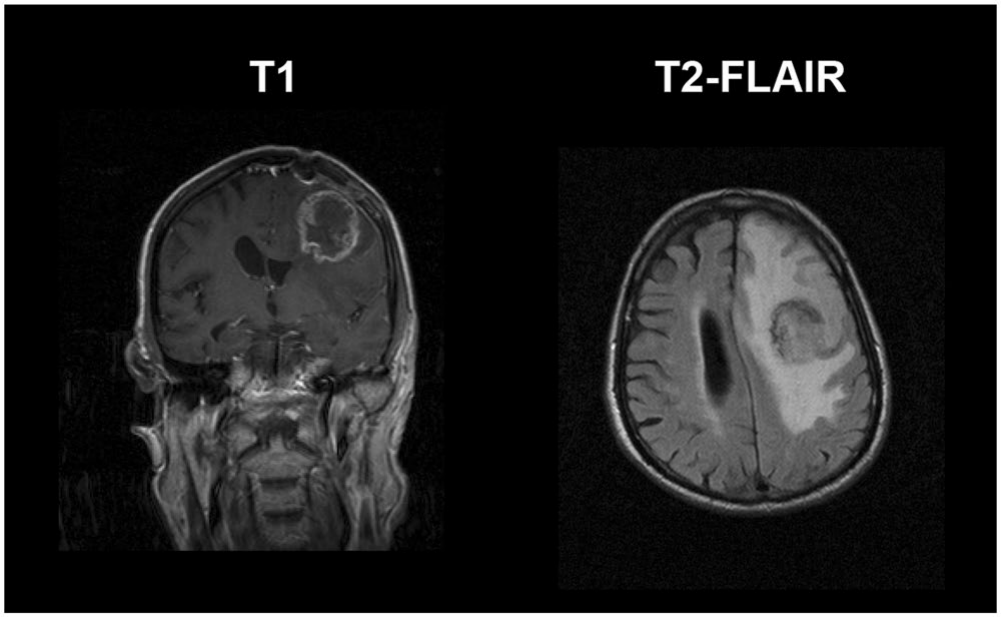
Post-biopsy Magnetic Resonance (MR) images from a 76-years-old male GB patient. On the left, a coronal T1-MR image, where the highly necrotic ring-shaped lesion can be seen frontotemporally in the left hemisphere, near motor area. Notice the mark of biopsy cavity formed by the trepanation of the skull. On the right, an axial T2-FLAIR MR image is shown, where the bright peritumoral infiltrative edema is remarkably extended causing space-occupation of the left hemisphere and internal pressing of the lateral ventricle.

In consultation with the Neurosurgical Clinic of the General University Hospital of Heraklion, Crete, Greece, patient-derived GB cells were collected during the biopsy of a patient with GB symptomatology (see Fig. 7) and no previous cancer record, while still naive from treatment. Small samples of different, non-necrotic, tumour regions were obtained and immediately transferred to cool sterile normal saline solution. Subsequent histopathological results positively confirmed the GB case. The cancerous tissue sample was anonymously provided with the informed patient’s written consent pre-operatively. All procedures and protocols follow the guidelines and have been approved by the Hospital’s Scientific Committee (Protocol number: 442120205-2018).

### Primary cell culture establishment

The tissue was directly transported to the lab, mechanically dissociated and cultured in serum-free Dulbecco’s modified Eagle medium (DMEM-F12) supplemented with cytokines (FGF2-EGF, Peprotech, UK) and B27 (ThermoFisher Scientific, UK), 10% of foetal bovine serum (FBS) and 50 μg/ml gentamycin (PANREAC Applichem, Germany). Aliquots at this stage (zero stage) were treated in order to preserve conditional stability and support the GB cells’ survival, aggregation and proliferation. Aliquots of zero stage, as well as various passages of *in vitro* cell tissue were cryopreserved, nucleic acids were extracted for further analysis and immunohistopathology was performed. The average doubling time intervals were estimated following a simple protocol in adherent cultures for both the established primary GB culture and the U87MG cell line which was used as a control (as described in^64^). All possible steps and procedures have been approved by the Institutional Ethical Committees.

### Invasion assay

GB spheroids were generated using the hanging-drop technique. The 3D spheroids were formed in a Perfecta3D 96-well hanging drop plate (3D Biomatrix, USA) by seeding a single cell suspension solution of approximately 600 cells/50 μl of supplemented DMEM per well for each cell type used. An agarose solution of 1% w/v was added to the plate’s reservoirs to prevent evaporation of the droplets.

After 4 days of spheroid formation, twenty spheroids per each cell type were transferred to a 96-well U-bottom plate, initially cooled on ice for 15–20 minutes. The invasion solution was made by diluting ice-cold BME Pathclear (Basement Membrane Extracts, Amsbio, Cultrex^®^, UK) in supplemented DMEM in a 1:1 ratio. In the U-bottom plate, 100 μl of the invasion solution was added per well containing either a primary or a U87MG spheroid. Subsequently, the U-bottom plate was centrifuged for 5 minutes at 300 rpm, at 4 °C in order to place the spheroids in the centre of each well, homogeneously distribute the invasion matrix and eliminate bubbles within it. Incubation for 1 hour at 37 °C was followed to allow solidification of the matrix. As a final step, 100 μl of warm supplemented DMEM was added per well and the plate was placed at a 37 °C humidified cell culture incubator to promote invasion to the semi-solid gel-like ECM matrix.

### Negative control

As a negative control experiment, spheroids of each cell line were examined by means of growing in the absence of the ECM-like substrate (i.e. in supplemented DMEM-F12 alone). It should be noted that none of the cell lines used exhibit invasion in the absence of ECM and no exogenous ECM is required for the spheroid formation via the hanging drop technique.

### Image segmentation and analysis

Spheroids were monitored using a Leica DFC310 FX inverse wide-field fluorescence microscope (Leica, Germany) over a total period of up to 12 days and photographed every 24 h, using a 4x objective lens and fixed acquisition parameters. The brightfield images were semi-automatically segmented in Matlab 6.1 (The MathWorks Inc., Natick, MA, USA).

Tumour expansion kinetics were evaluated based on: i) the time evolution of the tumour spheroid core, and ii) time evolution of the overall invasive rim^65^. The whole invasive area was measured by estimating the maximum radius taken from the core centre that encloses all the invasive cells. To estimate the invasive rim, the radius of the core maternal spheroid was subtracted from the whole invasive radius. The invasive kinetic profile was quantitatively generated by statistically analysing all results over time with regression analysis of mean values ± standard deviation.

### Mathematical approach

In cellular automaton (CA) models, each tumour cell operates individually (i.e. grows, divides, moves and dies) and interacts locally with other neighbouring cells following a set of biologically-inspired rules. CA models have been also extended to hybrid discrete-continuous (HDC) models in an attempt to additionally describe the interactions between cells and the microenvironment. These models integrate data from both experimental and/or clinical sources and have been widely used to describe critical aspects of tumour evolution and invasion, including genotype to phenotype relations^66^, inter- and intra-tumoral heterogeneity^19,67^, the effect of autocrine/paracrine signalling on cell proliferation and motility^66,68^, cell-to-cell and cell-to-matrix adhesion^33,51,67,69,70^, phenotypic plasticity^71–73^, the formation of invasive branches^74^, evolutionary dynamics^75,76^, the interplay with the brain anatomic features^77,78^ and the microenvironmental factors^79^, as well as treatment outcomes^80,81^.

In this work, we build on the HDC model originally proposed by Anderson^33^, but modify several aspects. Specifically, in order to focus on cell-to-cell adhesion, we consider the ECM to be a homogeneous passive scaffold where cells are allowed to migrate, but matrix degradation and remodelling are not considered. In our HDC approach, the phenotypic properties of the tumour cells include proliferation, motility, cell-to-cell adhesion, oxygen consumption and death. We assume that cell properties are intrinsic properties that are not regulated by the microenvironment. We account for heterogeneous cell populations, which differ only with respect to cell-to-cell adhesion properties. The rest phenotypic properties of the cells are kept the same for all cells, unless otherwise stated. The cell adhesive property is applied during cell movement and generalizes the attractive rule used in Aubert *et al*.^70^. Specifically, this property describes a cell’s preference to bind with a variable number of other cells in its new position. Thus, cells select their preferred neighbourhood as they move. Cells with low cell-to-cell adhesive properties prefer empty neighbourhoods, whereas cells with high adhesive properties are attracted towards highly populated areas. Cell movement approximates a random walk in a 2D regular lattice, but it is biased towards the adhesion preference of the cell. Cell division is a fundamental process that may change cell phenotype based on genetic, epigenetic, and/or stochastic decisions. If explicitly stated, inspired by its biological counterpart, we additionally introduced an intrinsic state transition probability, where cells are allowed to stochastically switch phenotype regarding cell-to-cell adhesion only during proliferation and with probability $\,{p}_{mut}$. Otherwise it is assumed that the adhesive property is inherited by the daughter cells during proliferation and it is fixed throughout tumour evolution. We assumed oxygen to be the only limiting source needed by the tumor cells to grow.

Cell processes are updated asynchronously and randomly (see Supplementary Text: *In silico* methods). This ensures that in each iteration every cell arbitrarily receives a different priority in the update queue. Cell movement and cell life cycle (including proliferation and death) are sequentially executed every $\,{t}_{r}=0.8\,h$. A more detailed description of the methodological approach follows.

### Computational domain

A 2D regular lattice of size L =5 mm was used to represent the computational domain. The 2D computational domain represents a planar slice through a 3D spheroid. Each $h\times h$ lattice site can accommodate only a single cell and its size was assumed to be equal to h = 20 μm.

### Cell proliferation

The proliferation age of tumour cells was approximated by the relevant doubling time biological experiments. To proliferate, cells must find empty space for their daughter cells. Otherwise, the cell enters a quiescent state, while it keeps searching for empty space. If a quiescent cell finds an empty space, it immediately proliferates. The neighbourhood chosen for the proliferation was the Moore neighbourhood of size r equal to 2 (for more details also see^64^).

### Cell movement

In general, tumour cell motility involves highly complex mechanisms, yet for simplicity and in an attempt to focus on cell adhesion; we only assumed random, diffusive movement and accounted for cell-to-cell adhesion forces. Cells are allowed to move towards empty neighbouring locations in the Moore neighbourhood. The diffusion equation (1) is discretized to movement probabilities for each individual cell, as has been described in^82^. In

1 $$\frac{\partial c}{\partial t}={D}_{c}{\nabla }^{2}c,$$

$c$ and ${D}_{c}$ denote the cancer cells concentration and their diffusion coefficient, respectively.

**Figure 8:**
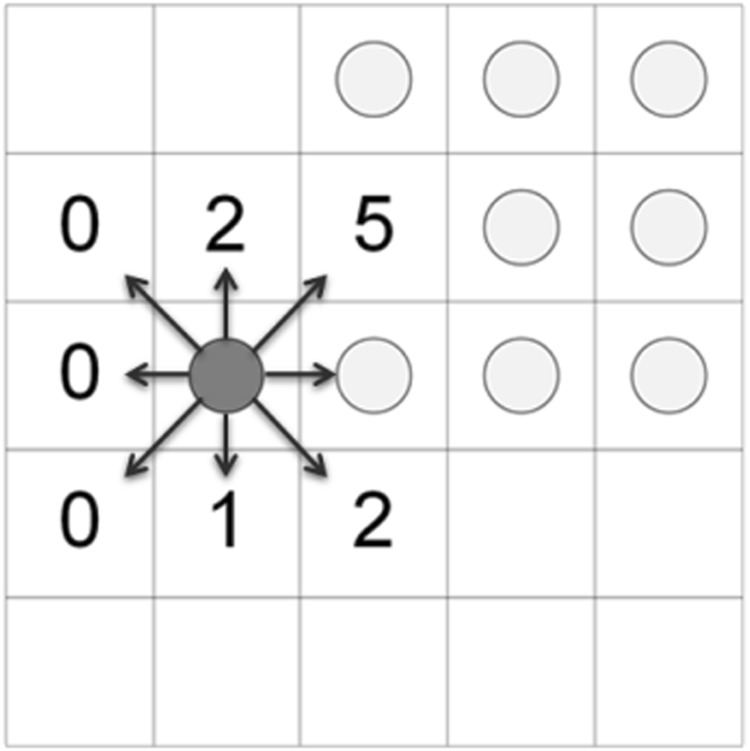
Cell movement depending on adhesion preference. Circles represent cancer cells. The dark grey circle depicts the cancer cell, under investigation. The numbers depict the occupation of each neighbourhood, excluding the cell under study. The numbers can thus take values between 0 (non-populated area) and 7 (highly populated area). Cells move to empty sites depending on their adhesion preference. Thus, if for example the cancer cell under study has adhesion preference equal to 0, then it will move left, randomly selecting one of the three possible positions.

The mechanism of adhesion preference is formulated as follows: a cell will only move to empty adjacent locations with neighbours equal to its adhesion preference, which can vary between 0 (non-populated area) and 7 (highly populated area). Schematically, cell movement under the inclusion of cell adhesion preference is shown in Fig. 8.

### Cell Death

Lack of oxygen triggers cell death. The spatiotemporal evolution of oxygen ($o$) is described in

2 $$\frac{\partial o(x,y,t)}{\partial t}={D}_{o}{\nabla }^{2}o(x,y,t)-{\gamma }_{o}o(x,y,t){c}_{i,j}-{\alpha }_{o}o(x,y,t).$$

Oxygen diffuses with diffusion constant $\,{D}_{o}\,\,$from the boundaries of the computational domain, naturally decays at rate $\,{\alpha }_{o}$ and is consumed by the tumour cells at rate $\,{\gamma }_{o}$. The term ${c}_{i,j}\in \{0,1\}$ indicates the presence or not of a tumour cell at the lattice point $i,j$. In order to mimic the laboratory conditions of the medium, oxygen concentration was set to its maximal value at the edge of the computation domain through the application of Dirichlet boundary conditions.

Tumour cells die if the local oxygen concentration drops below ${o}_{deadly}$. Dead cells are essentially treated as empty space.

### Description of phenotypes

Phenotypes with different adhesion preferences were allowed to coexist and interact within the tumour. The different phenotypes are referred based on their preference adhesion value. A phenotype with low adhesion value corresponds to a cell with loose cell-to-cell adhesive interactions that prefers to be alone, while a phenotype with high adhesive value implies that a cell forms strong adhesive interactions, attracted by high populated neighbourhoods. We categorize our phenotypes as follows: we call phenotypes with adhesion preference 0 and 1, low adhesive; phenotypes with preference 6 and 7, highly adhesive; and those with adhesion preference in [2, 5], middle adhesive phenotypes.

## Electronic supplementary material

Supplementary information

SV1

SV2

## Data Availability

All data generated and analysed during this study are included in this published article (and its Supplementary Information files).
