# Supplementary material for: Integrating in vitro experiments with in silico approaches for Glioblastoma invasion: the role of cell-to-cell adhesion heterogeneity
Source: Sci Rep. 2018 Nov 1;8:16200. doi: 10.1038/s41598-018-34521-5 (PMC6212459; doi:10.1038/s41598-018-34521-5)
Supplement: Supplementary file 1 — Supplementary information [file 41598_2018_34521_MOESM1_ESM.pdf]

Supporting information

Integrating *in vitro* experiments with *in silico* approaches for Glioblastoma invasion: the role of cell-to-cell adhesion heterogeneity

Oraiopoulou M-E [1,2], Tzamali E [2], Tzedakis G [2], Liapis E [3,#a], Zacharakis G [3], Vakis A [1,4], Papamatheakis J [5,6], Sakkalis V [2,*]

[1] Department of Medicine, University of Crete, Heraklion, Crete, Greece
[2] Computational Bio-Medicine Laboratory, Institute of Computer Science, Foundation for Research and Technology-Hellas, Heraklion, Crete, Greece
[3] Institute of Electronic Structure and Laser, Foundation for Research and Technology-Hellas, Heraklion, Crete, Greece
[4] Neurosurgery Clinic, University General Hospital of Heraklion, Crete, Greece
[5] Gene Expression Laboratory, Institute of Molecular Biology and Biotechnology, Foundation for Research and Technology-Hellas, Heraklion, Crete, Greece
[6] Department of Biology, University of Crete, Heraklion, Crete, Greece
[#a] Current address: Helmholtz Zentrum München, German Research Center for Environmental Health (GmbH), Neuherberg, Germany[*] Correspondence to [sakkalis@ics.forth.gr]

In silico methods

A hybrid discrete-continuous computational model was used to explore the particular influence of cell-to-cell adhesive heterogeneity on the formation of invasive morphologies and reproduce our *in vitro* experimental observations and their tumor kinetics. The model thus consists of two interacting compartments; the discrete reflecting the tumor cells and the continuous one representing the tumor micro-environment. A 2D regular lattice represents the computational domain. A lattice site can accommodate only a single cell. The flow chart below (Figure A) briefly shows the function of the discrete compartment. The discrete compartment regards the cell's life cycle and particularly the cell proliferation and death. We considered the extracellular matrix (ECM) a homogeneous passive scaffold where cells are allowed to migrate but, matrix degradation and remodeling are not taken into account. Random, diffusive cell movement biased towards the adhesion preference of the cell is assumed. The mechanism of adhesion preference is formulated as follows: a cell only moves to empty adjacent locations with neighbors equal to its adhesion preference, which can vary between 0 (non-populated area) and 7 (highly populated area) in Moore neighborhood. The different phenotypes are referred based on their preference adhesion value. We further assumed oxygen to be the only limiting source needed by the tumor cells to grow. A continuous reaction-diffusion equation describes the spatiotemporal evolution of oxygen.

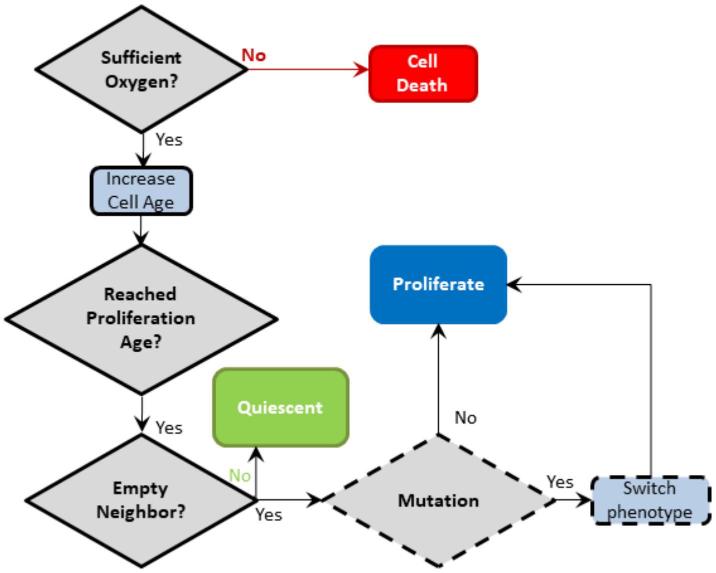

Figure A: Flow chart displaying the discrete cell life and the cell state transitions.

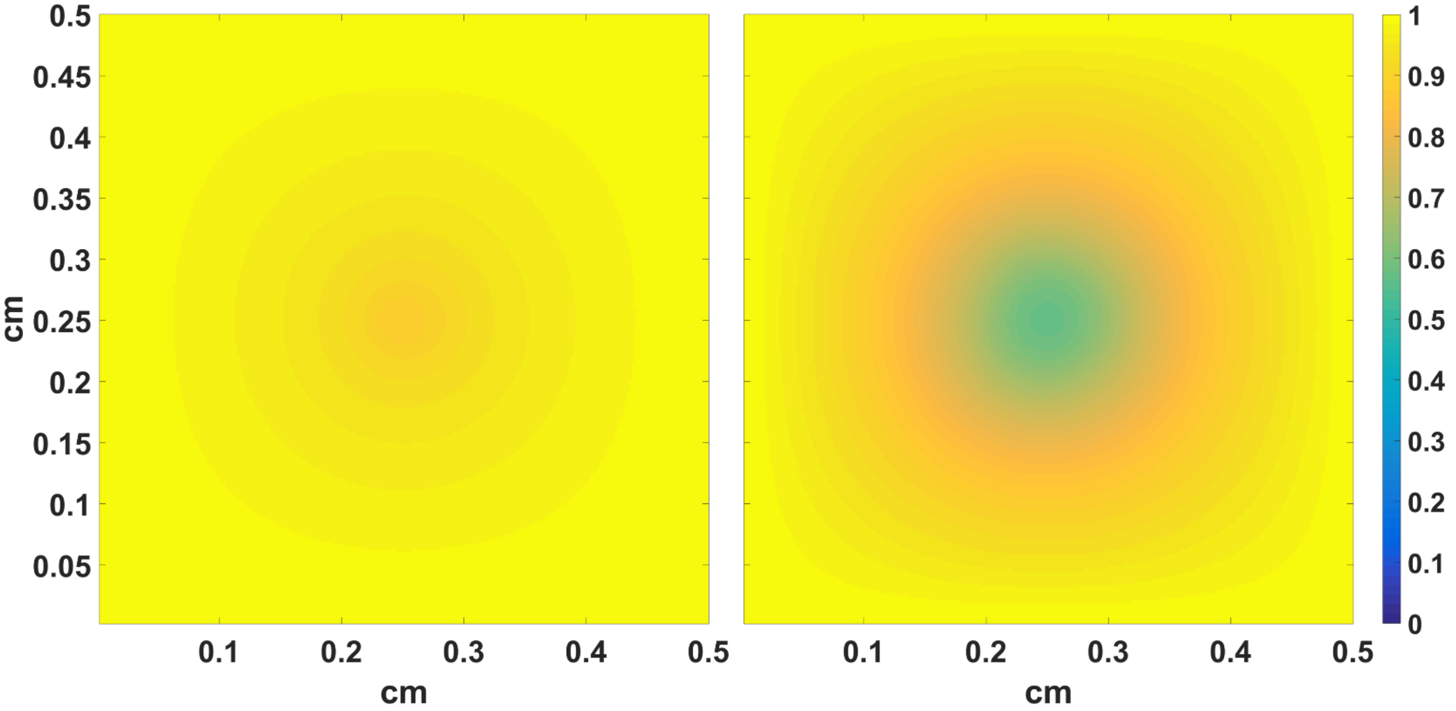

Figure B: Oxygen concentration of the main simulations for U87 (left) and P03 (right) at 96 hours.

Tumor cells die if the local oxygen concentration drops below a random threshold. Specifically, the threshold is assumed to be $o_{deadly} \cdot (1 + r)$, where r , a random variable from the uniform interval, is $[-0.5, 0.5]$ and o_{deadly} is as shown in Table 1. The choice of the oxygen threshold ensures that its mean value is o_{deadly} over time and that cell death is not synchronized. A similar technique is used to ensure that proliferation is not synchronized while ensuring that on average the proliferation rate is unaffected. After reaching their maturity age, in order to proliferate, cells must find empty space for their daughter cells. If no empty space is found in the neighborhood, the cell enters a quiescent state while it keeps searching for empty space. The parameters used in the computational simulations are presented in Table 1 and Table 2. We investigated: i) the case where no phenotypic switch is allowed in cell populations and all cell properties are inherited to the daughter cells during proliferation and remain fixed throughout tumor evolution and ii) the case where cells are allowed to randomly switch between phenotypes with equal probability. The U87MG cells were allowed to switch to phenotypes 0, 1, 6 and 7 while the possible phenotypes for the primary cell line were 2 through 7. Phenotypic switch regards cell-to-cell adhesion, only during proliferation and with probability p_{mut} equal to 0.5.

Several additional computational experiments are presented in the following. Specifically, we investigate the effect of proliferation and motility rates on tumor expansion and morphology as well as the role of phenotypic switch on tumor evolution. Due to the stochastic components in the approach, we repeated each experiment 5 times for the additional experiments shown here and depict the average and standard deviation values. A spectrum of morphologies that can arise from the combination of phenotypes with different adhesive properties is also shown.

Simulation pseudocode

- Initialize continuous variables and cells
- **iteration** = 0
- while **iteration** < total_iterations and cells have not reached the edge of the domain
  - **t** = 0
  - while **t** < τ
    - Update continuous environment
    - Execute cell life (see chart): kill cells, increase age, proliferate cells, etc.
    - Execute cell movement
    - **t** ← **t** +t_r
  - **iteration**++

Simulation pseudocode

Table 1: General in silico parameters.

**Domain size L**	$5\ mm$
**Iteration length τ**	$8\ h$
**Deadly oxygen threshold o_{deadly}**	0.2 (ND)
**Oxygen decay α**	0.0125 (ND)
**Oxygen consumption γ**	$6.25 \times 10^{-17}\ M\ cell^{-1}s^{-1}$
**Oxygen Diffusion D_o**	$10^{-5}\ cm^2/s$

Table 2: Cell line specific parameters.

	**U87MG**	**Primary**
**Phenotypes used**	0,1,6,7	2,3,4,5,6,7
**Proliferation age**	31 hours	25 hours
**Diffusion rates D_{c1}, D_{c2}**	$1.5 \cdot 10^{-8}, 3 \cdot 10^{-9}\ cm^2/s$	$4 \cdot 10^{-10}, 4 \cdot 10^{-8}\ cm^2/s$
**Diffusion rate D_c**	$5 \cdot 10^{-9}\ cm^2/s$	$2 \cdot 10^{-8}\ cm^2/s$
**Initial radius**	$140\ \mu m$ (7 cells)	$200\ \mu m$ (10 cells)

Quantitative metrics for evaluating in silico tumor morphology and expansion

Measuring Compactness and sparseness

In order to quantify how compact and sparse the resulting emerging morphologies are, to measure the compactness and sparseness we count the number of live cells in the 1-moore neighborhood for every lattice point, deriving the number of neighbors for every live cell. In Figure C we illustrate the result of measuring the sample morphologies from Figure 5a and Figure 6a of the main text. The relative cell populations with high and low neighbors are used to measure compactness and sparseness, respectively.

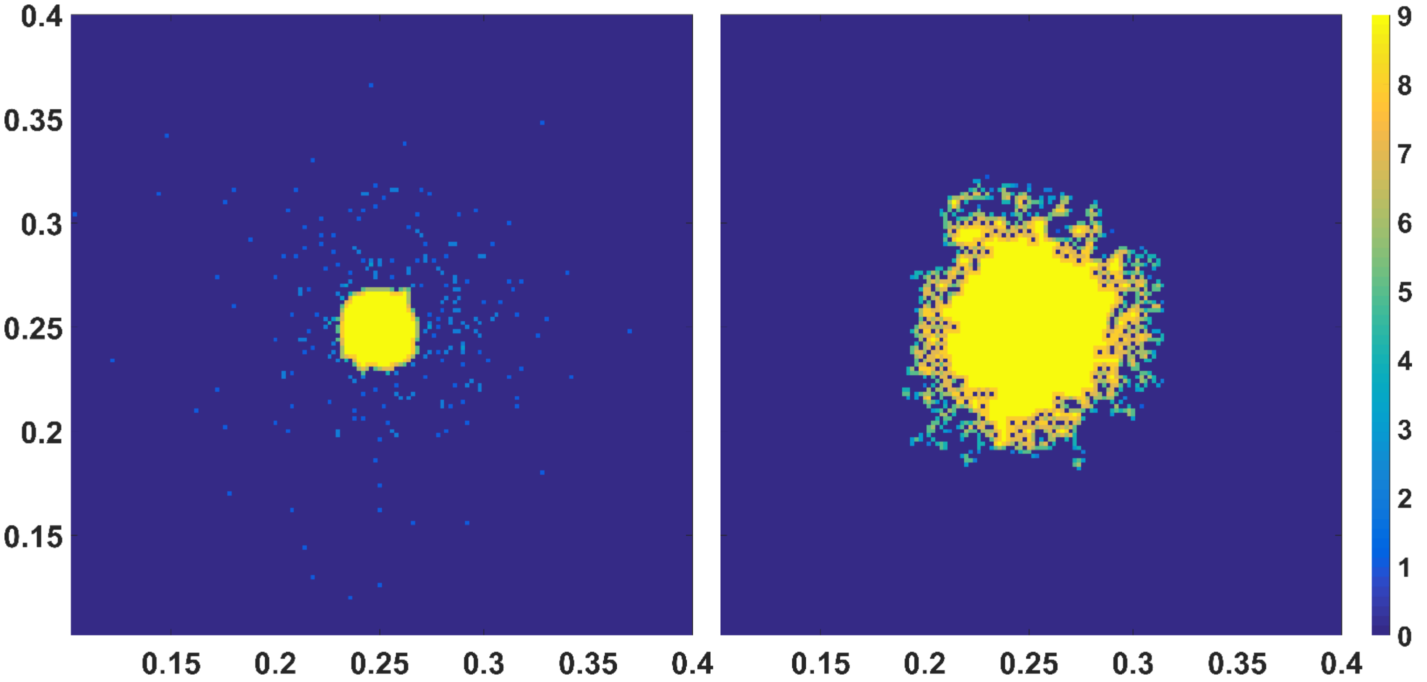

Figure C: Illustration of the number of live cells in every neighbor for the sample morphologies shown in the main text.

Different adhesion properties produce different morphologies

A spectrum of morphologies emerge when phenotypes with various cell-to-cell adhesion properties are combined. Phenotypes were separated in three groups based on their adhesion preference. Groups were named low, medium and high and correspond to {0, 1}, {2, 3, 4, 5} and {6, 7} adhesion preferences, respectively. The experiments were performed using the proliferation and diffusion rates of the U87MG computational experiments as shown in Table 2. The temporal evolution of the core and the invasive radii, the cell density profile as well as the local compactness and local sparseness of the tumor were estimated. Combinations of phenotypes in which low adhesive phenotypes are included show increased expansion of invasive rim and radius, increased sparsity and decreased compactness compared with the other combinations. Interestingly, the compactness of the middle adhesive phenotypes is initially decreased but at later time points it significantly increases approximating the compactness of the combined middle and highly adhesive phenotypes.

Single phenotypic group

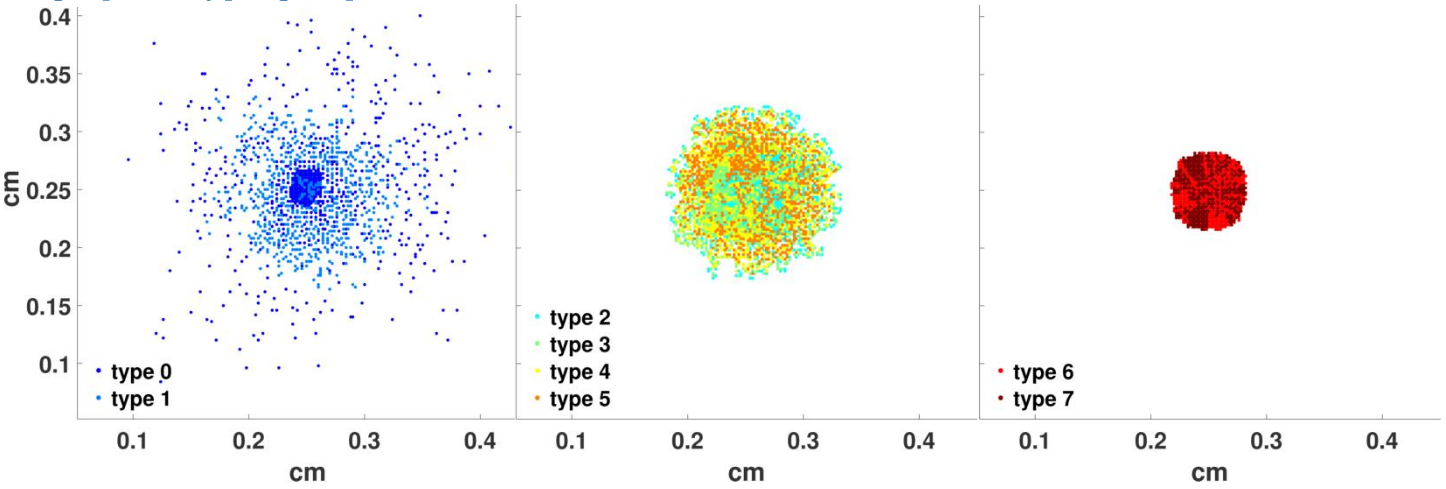

Figure D: From left to right, phenotypic groups of low, medium and high adhesion preference at 112, 216 and 216 hours, respectively.

Mixture of phenotypic groups

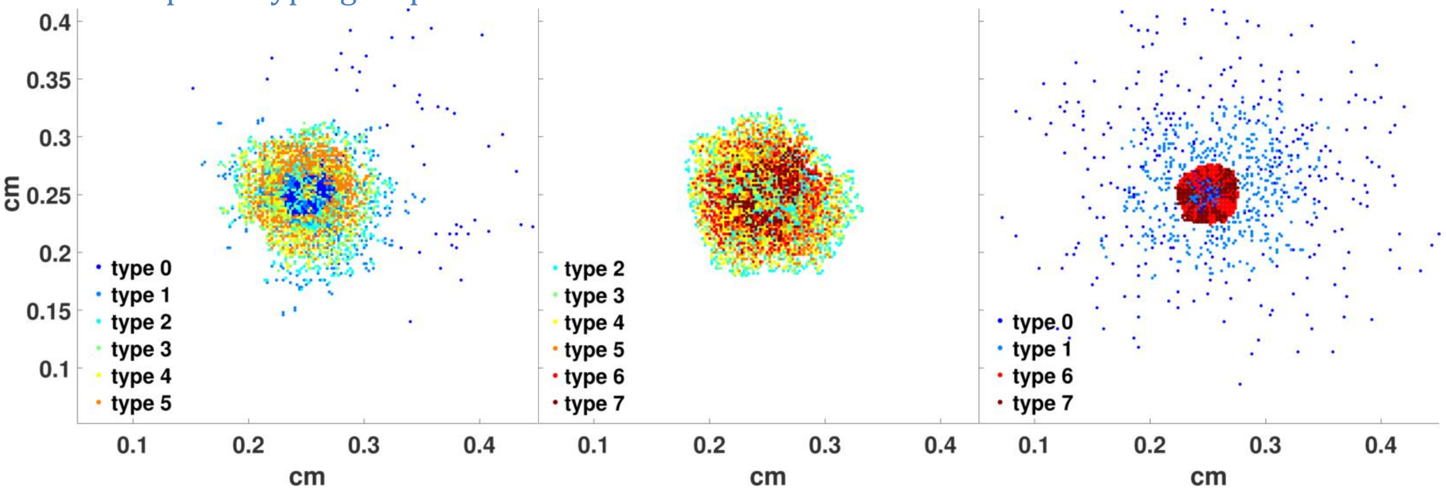

Figure E: From left to right, snapshots of phenotypic groups of {low, medium}, {medium, high} and {low, high} at 176, 216 and 160 hours are shown, respectively.

All phenotypic groups

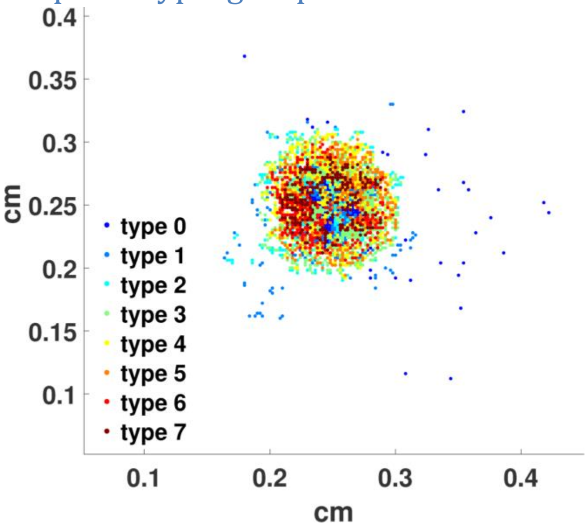

Figure F: Resulting morphology from combination of all possible adhesion preferences at 184 hours.

Subgroups of middle adhesive phenotypes

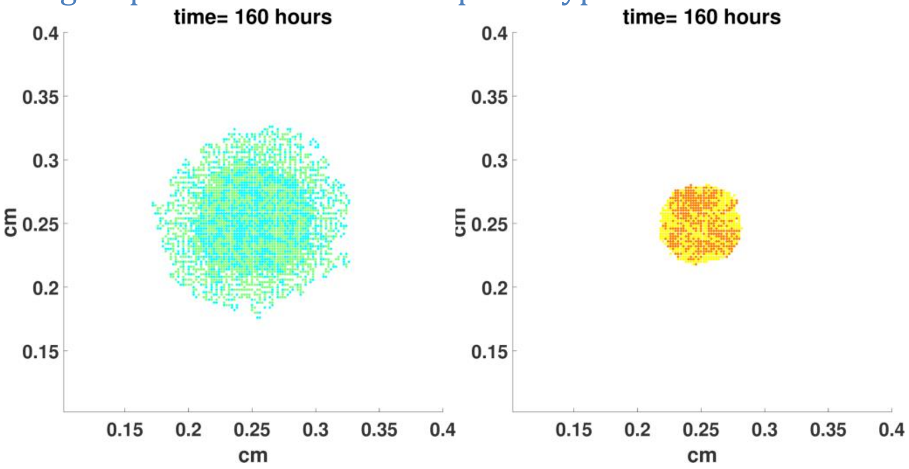

Figure G: Resulting morphology from combination of type 2 and 3 (Left) and type 4 and 5 (Right) at 160 hours.

Mixture of low with subgroups of middle adhesive phenotypes

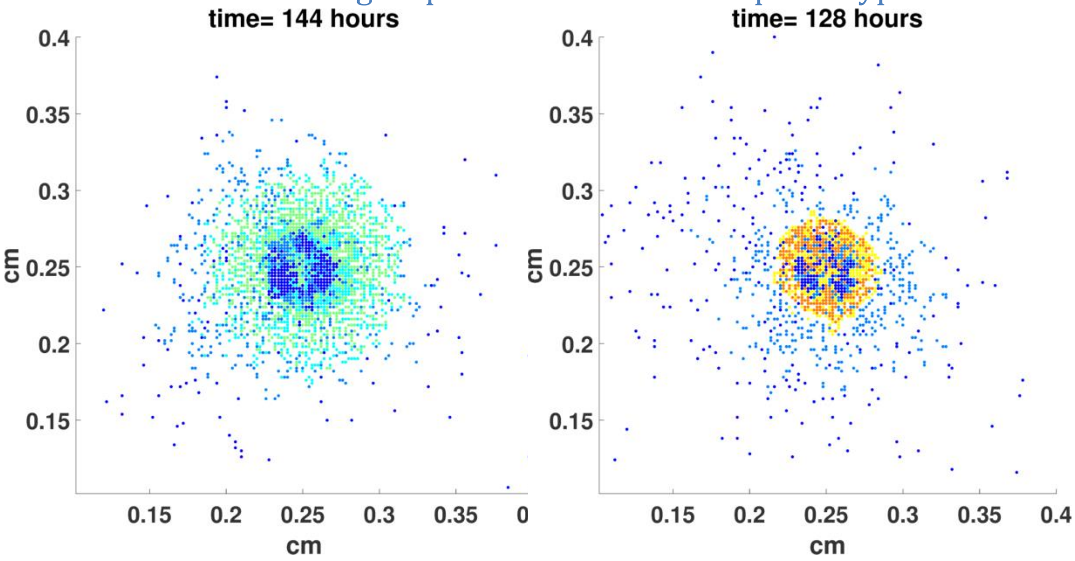

Figure H: Resulting morphology from combination of type 2 and 3 (Left) and type 4 and 5 (Right) with low adhesive phenotypes at 144 and 128 hours, respectively.

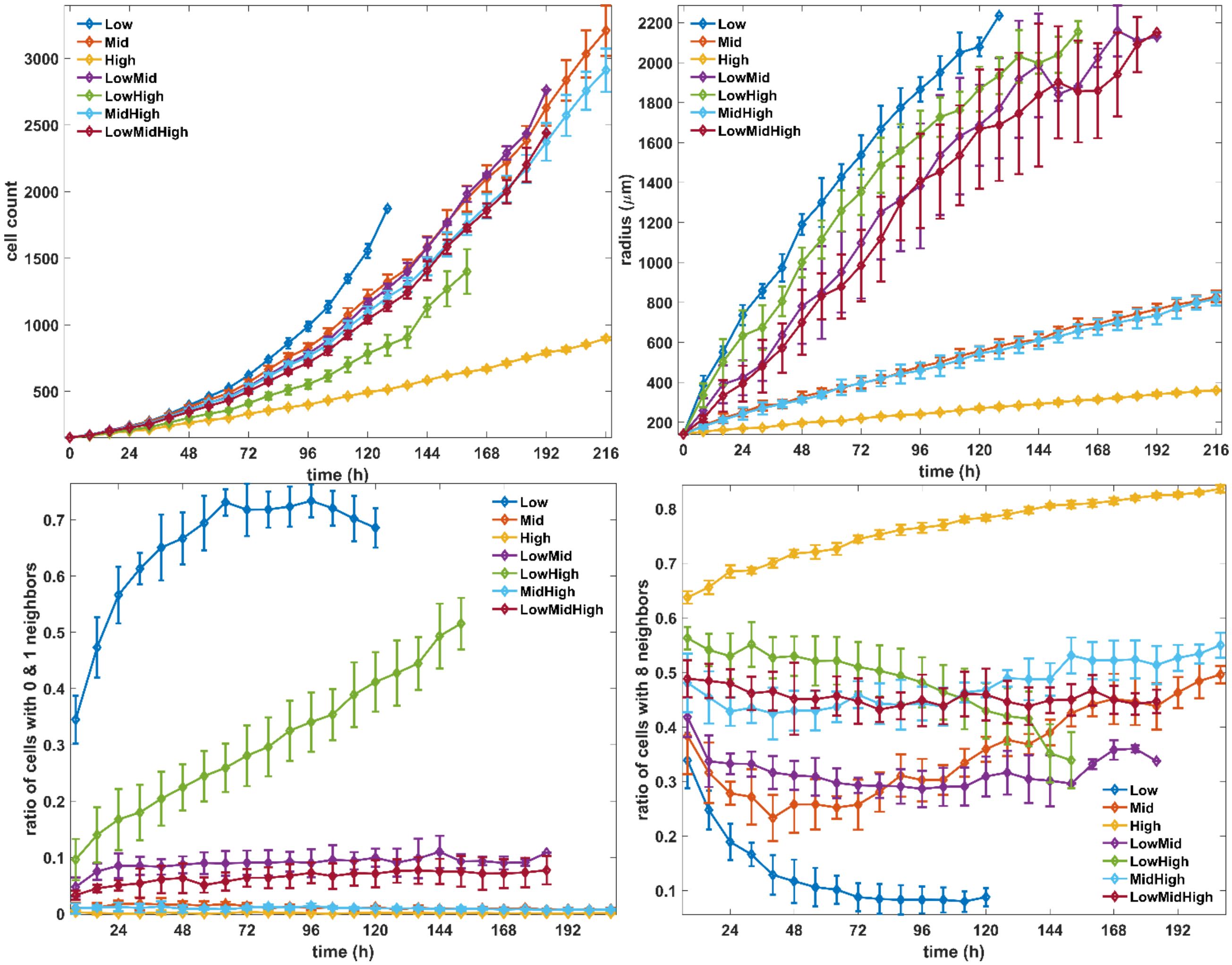

Figure I: Temporal evolution of cell population, invasive radius, the sparseness and compactness for different combinations of phenotypes. Data derived from 5 simulations per population setup.

In silico experiments timeline

U87MG spheroids

In order to describe the U87MG cell line, phenotypes with loose cell-to-cell adhesive interactions (types 0 and 1) and very strong adhesive interactions (types 6 and 7) were only considered. Snapshots of the simulated U87MG line for the time points observed in the *in vitro* data are shown. The simulated evolution of the U87MG invasive spheroids can be also seen in the Supplementary video SV1.

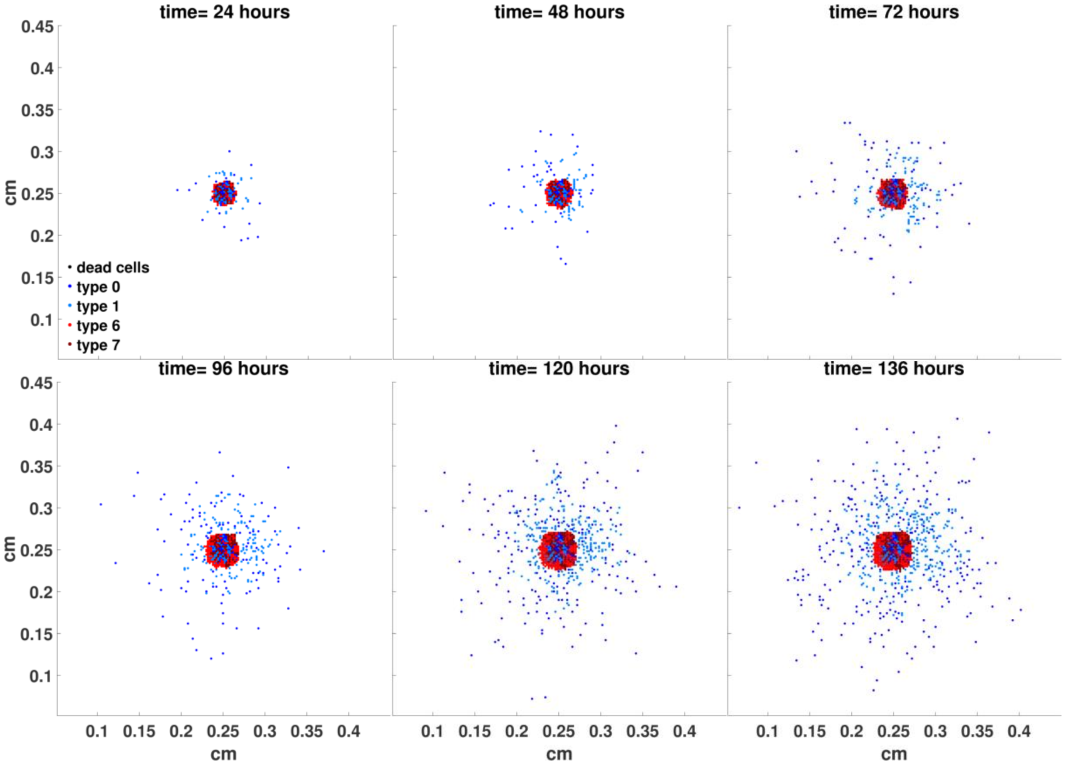

Figure J: Snapshots of the simulated U87MG line for the time points observed in the in vitro data. The experiment was termintated at 136 hours because invasive cells were in proximity of the domain edge.

Primary GB spheroids

In order to describe the primary GB invasive pattern, phenotypes with middle cell-to-cell adhesive interactions (types 2-5) and very strong adhesive interactions (types 6 and 7) were considered. Alternative combinations of phenotypes could also produce similar morphologic characteristics. For example, the pattern of the middle adhesive phenotypes is very similar with that of primary with respect to all the metrics we have used in this work including the cell population density, the invasive radius as well as the local sparseness as shown previously in Figure I. They differ only with respect to compactness. The middle adhesive phenotypes are initially considerably less compact than the primary (middle and highly adhesive phenotypes combined). After a period of time, they start becoming more compact and eventually reach the compactness of the primary cells. Advanced imaging modalities that could approximate the cellularity within a given tumor volume would aid in our understanding of pattern formation and the phenotypic heterogeneity involved. Snapshots of the simulated primary GB cells for the time points observed in the *in vitro* data are shown in Figure K. The simulated evolution of the primary invasive spheroids can be also seen in the Supplementary video SV2. The temporal evolution of phenotypes and the radial profile at 152 hours shown in Figure L illustrate the emerging spatial self-organization of the phenotypes and its effect on the sub-populations. Type 7 cells are eradicated due to the fact that they mostly reside closer to the core which also is where the necrosis starts to appear.

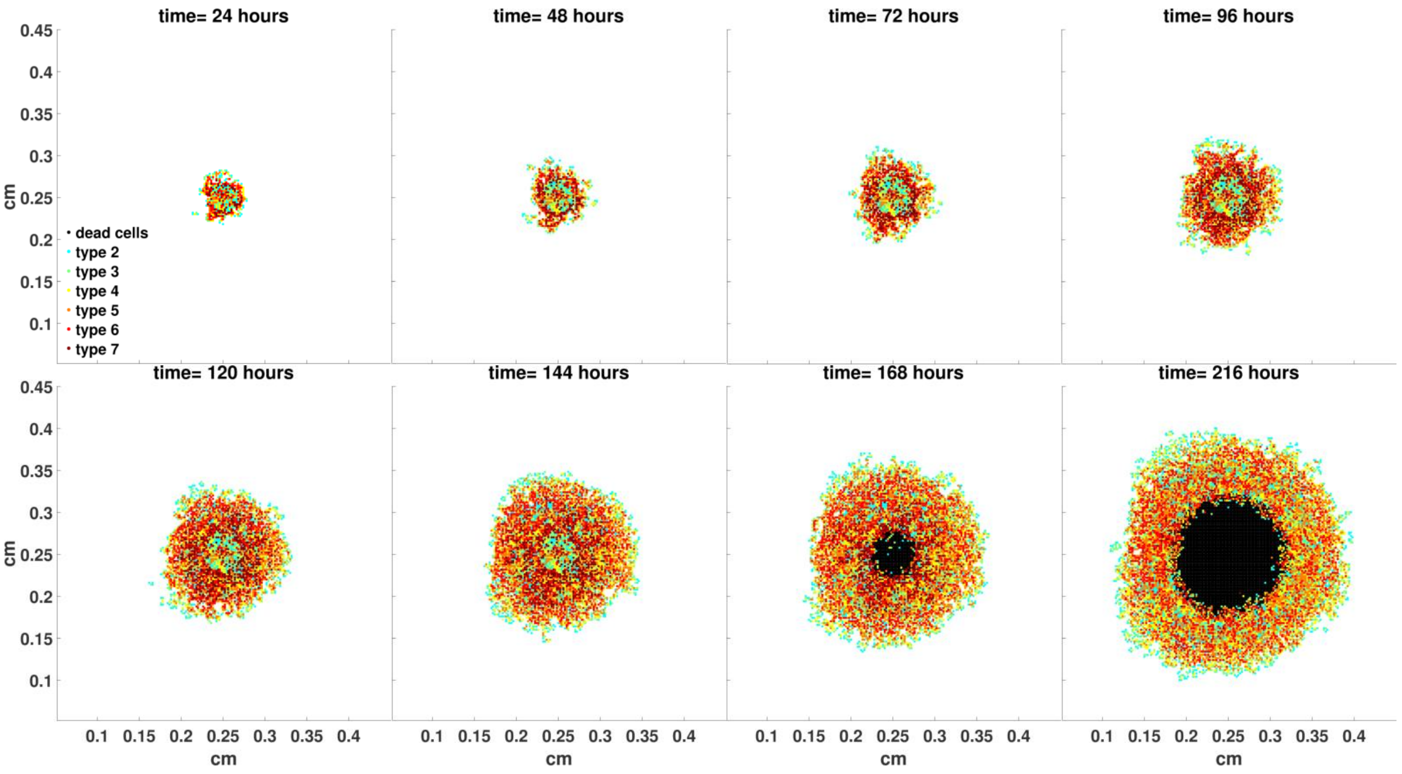

Figure K: Snapshots of the simulated primary GB cell line for the time points observed in the in vitro data.

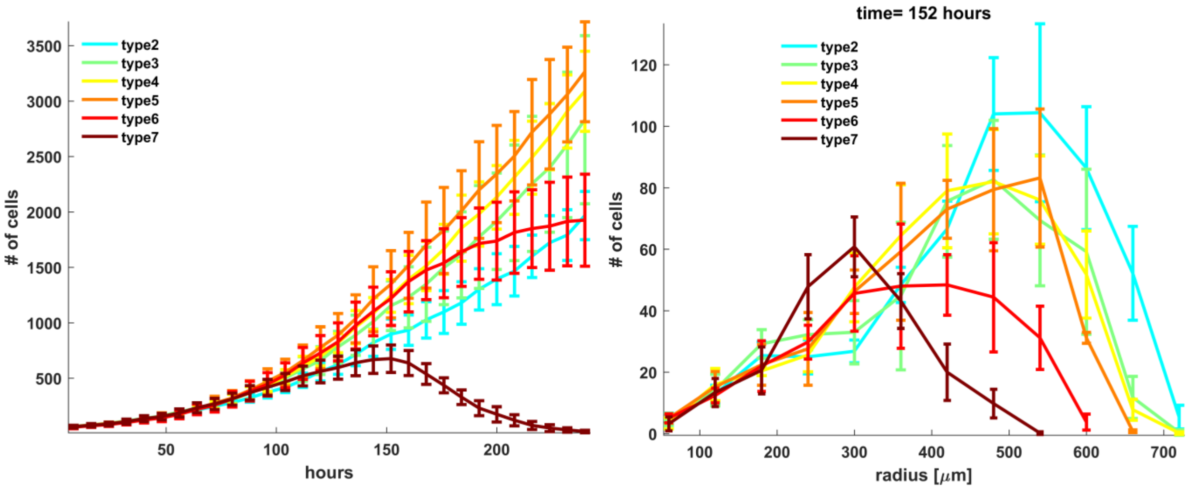

Figure L: Temporal evolution of phenotypes in the simulated primary GB cell line (left) and the radial distribution of phenotypes at 152 hours (right). Mean values and standard deviations were calculated by 50 simulations.

Effect of proliferation and motility rates on tumor morphology and expansion

Two different scenarios were explored for both cell lines. In the first, we alter the proliferation rate of all phenotypes involved and in the second, we selectively inhibit the proliferation of the low adhesive phenotypes for the U87MG cells and of the middle adhesive phenotypes for the primary cells, while the highly adhesive phenotypes keep proliferating.

U87MG cells

Proliferation

Proliferation affects the temporal evolution of cell population and affects both the expansion of the core radius and interestingly, the invasive radius. The effect of proliferation in invasive radius is particularly evident when we compare it with the second scenario where the highly adhesive phenotypes are only allowed to proliferate (yellow line relative to green line). Thus, a go or grow mechanism if applied would reduce the cell density and the invasive radius. The invasive radius as expected and we shall see next is affected by the motility rate, therefore increased motility could be assumed to compensate the inhibition of proliferation and produce similar dynamics.

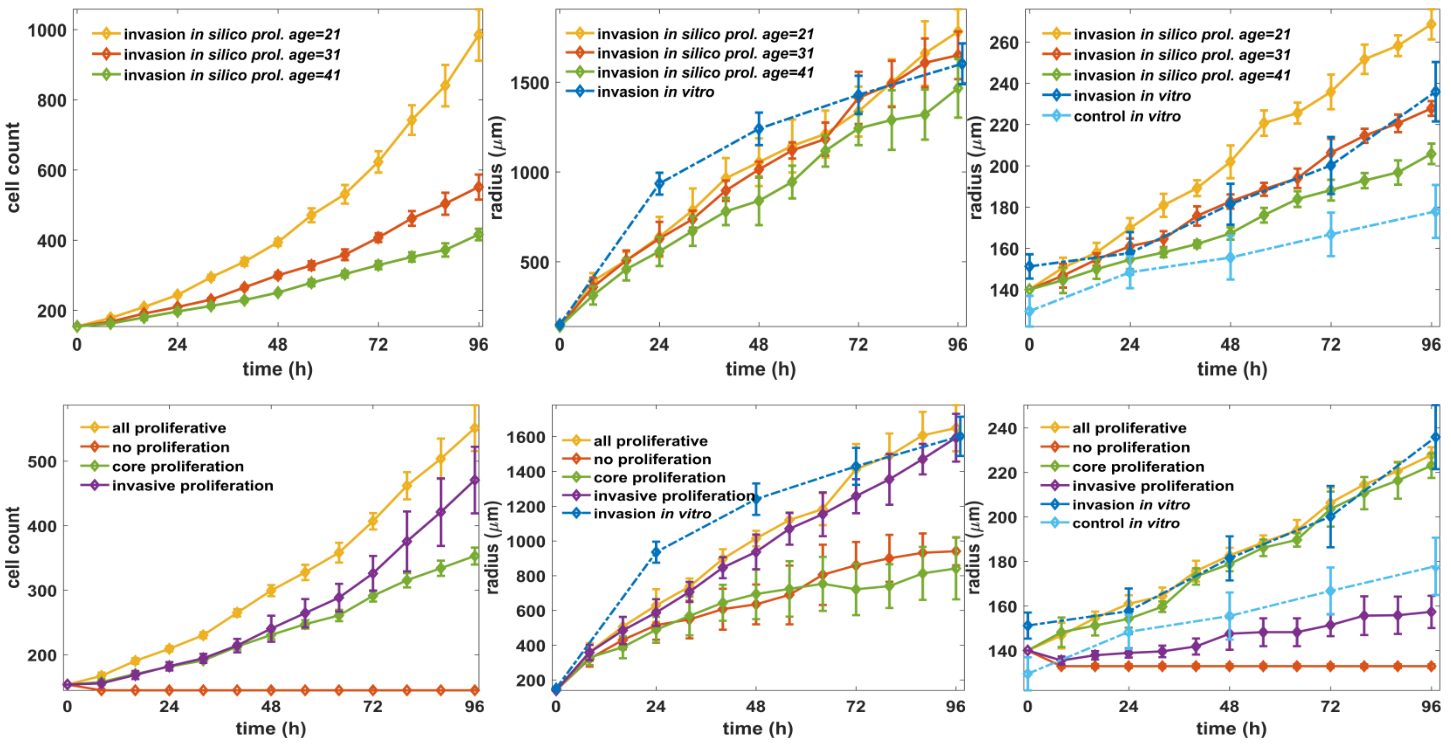

Figure M: Impact of the proliferation rate on the evolution of cell population, overall invasive area and tumor core of the U87MG spheroids.

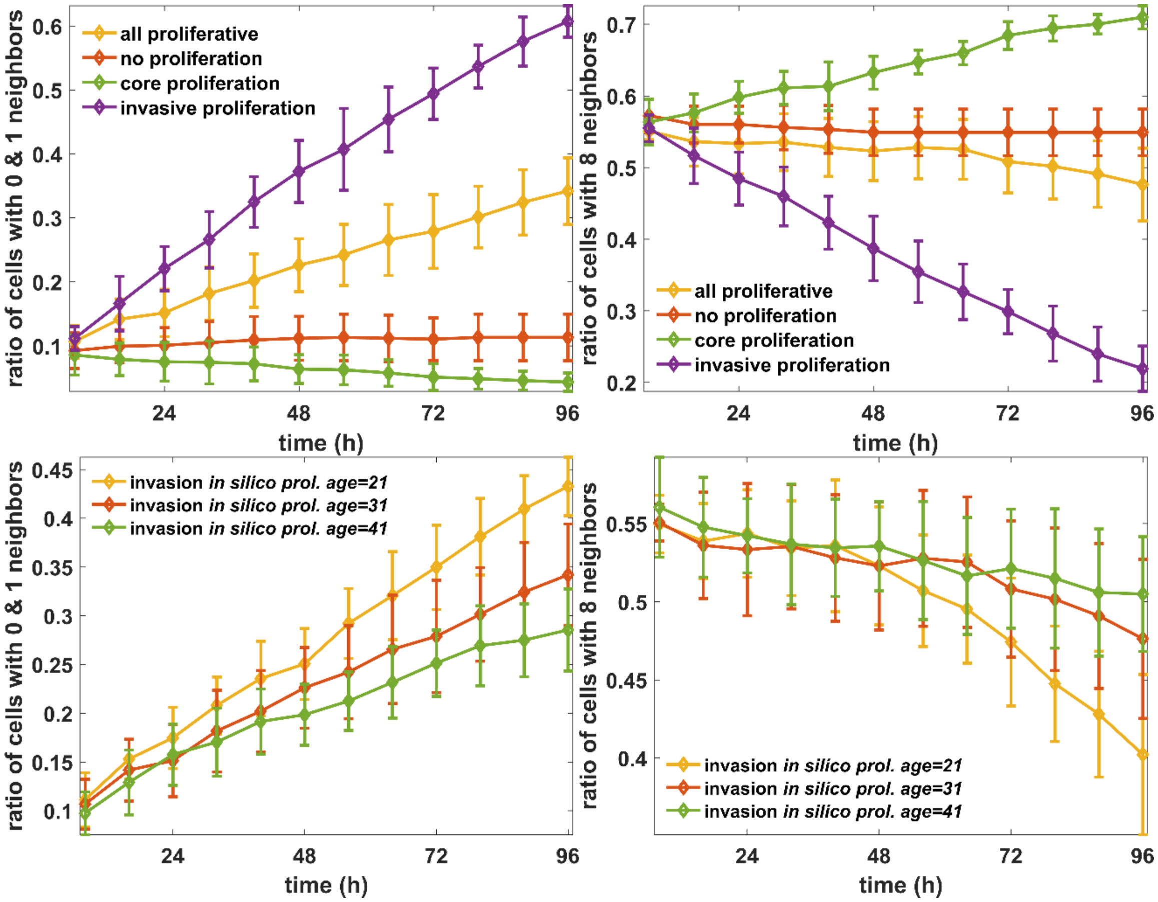

Figure N: Impact of proliferation on tumor sparsity and compactness.

Motility

Increased motility rate considerably increases the invasive radius but only slightly affects the expansion of the core and the cell population density.

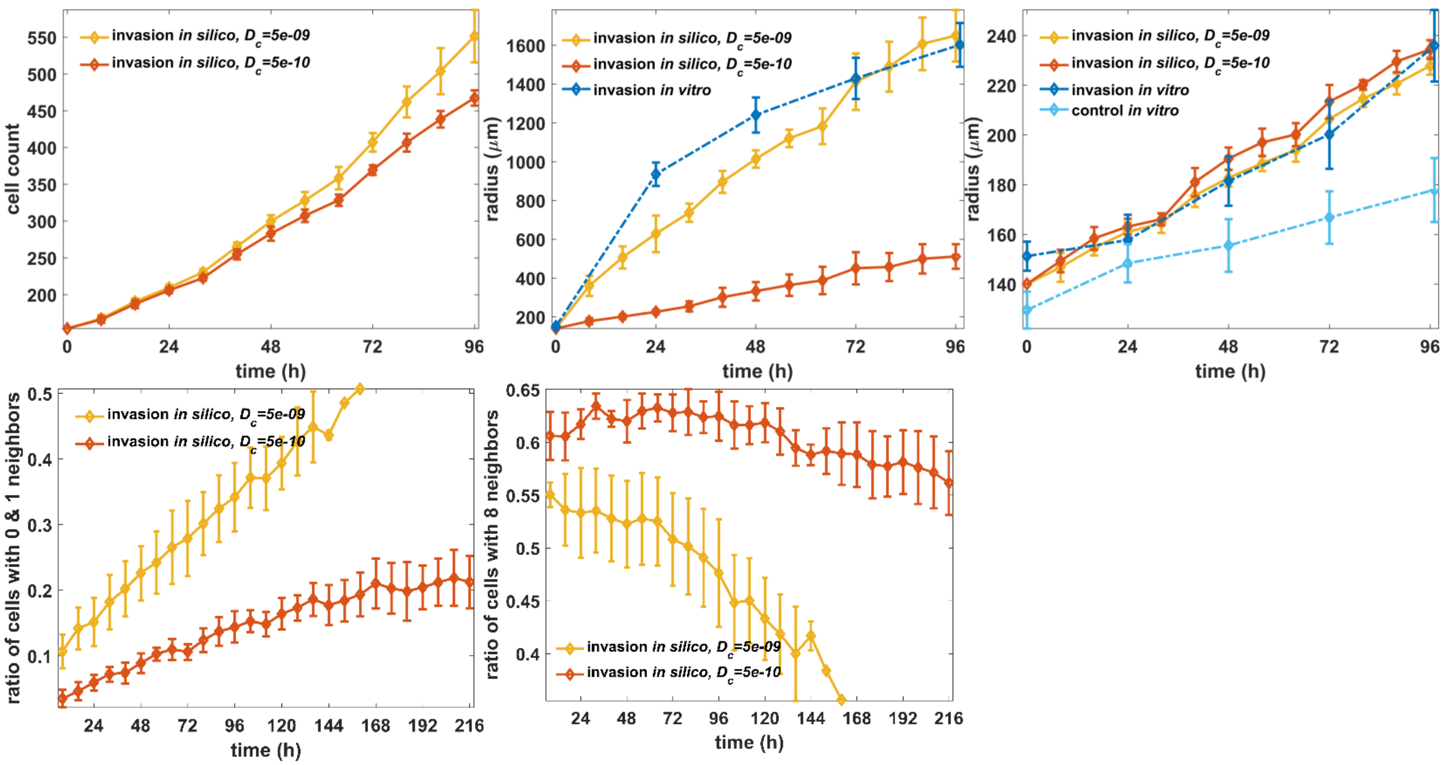

Figure O: Impact of the diffusion rate on the evolution of cell population, overall invasive area and tumor core of the U87MG spheroids.

Primary cells

Proliferation

Increased proliferation rate results in faster tumor expansion and higher cell populations. Increased proliferation also results in increase of the tumor compactness and reduces the sparseness at the tumor boundaries resulting in more smooth and round tumors. Selective proliferation of the highly adhesive phenotypes results in their dominance in the population and the formation of considerably dense tumors with no invasive rim and decreased overall cell population. Proliferation has thus a dramatic effect on morphology and expansion of primary cells mainly because it affects the relative ratio of the phenotypes involved.

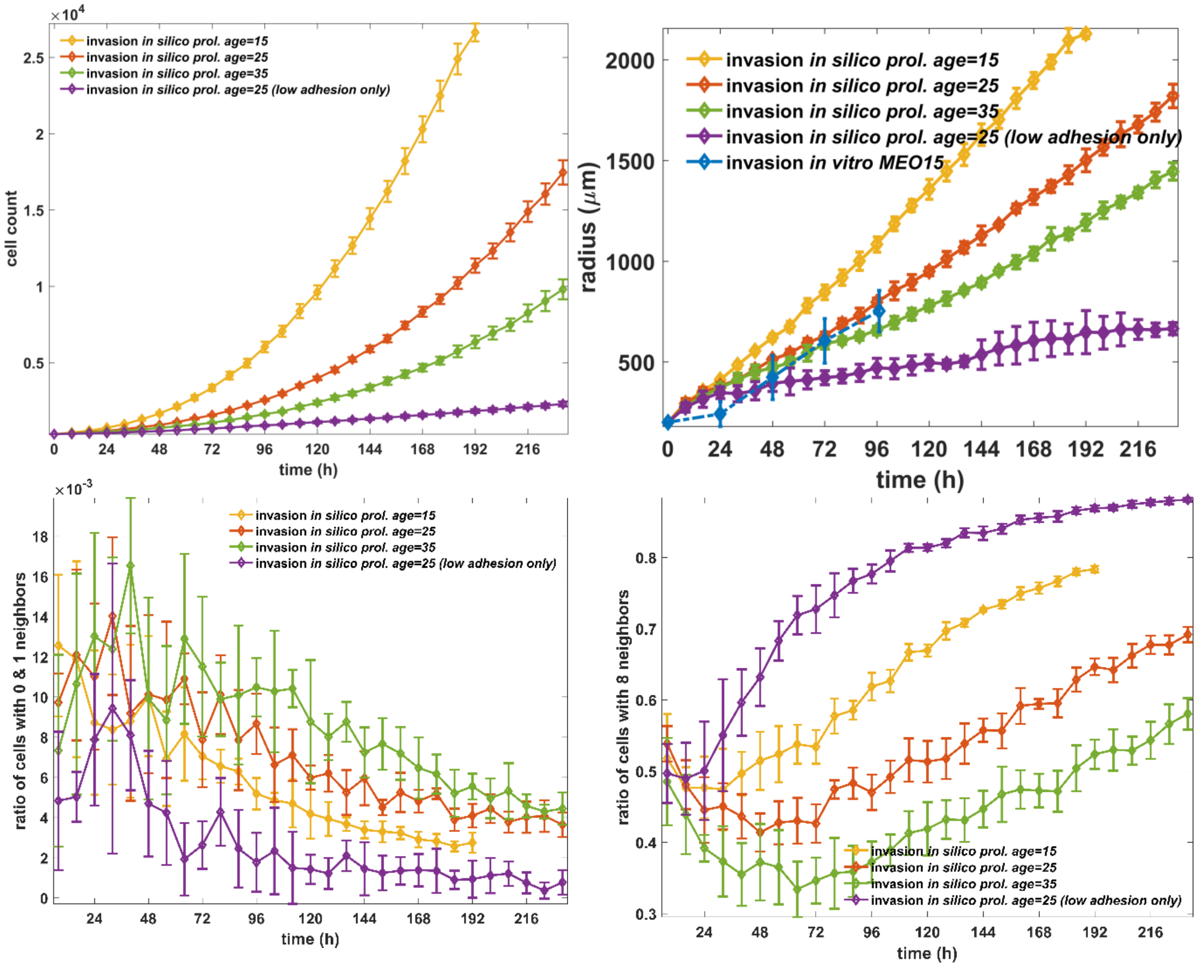

Figure P: Impact of the proliferation rate on the evolution of cell population (top left), overall invasive area (top right), the sparseness (bottom left) and compactness (bottom right) for different proliferation times of the primary GB spheroids.

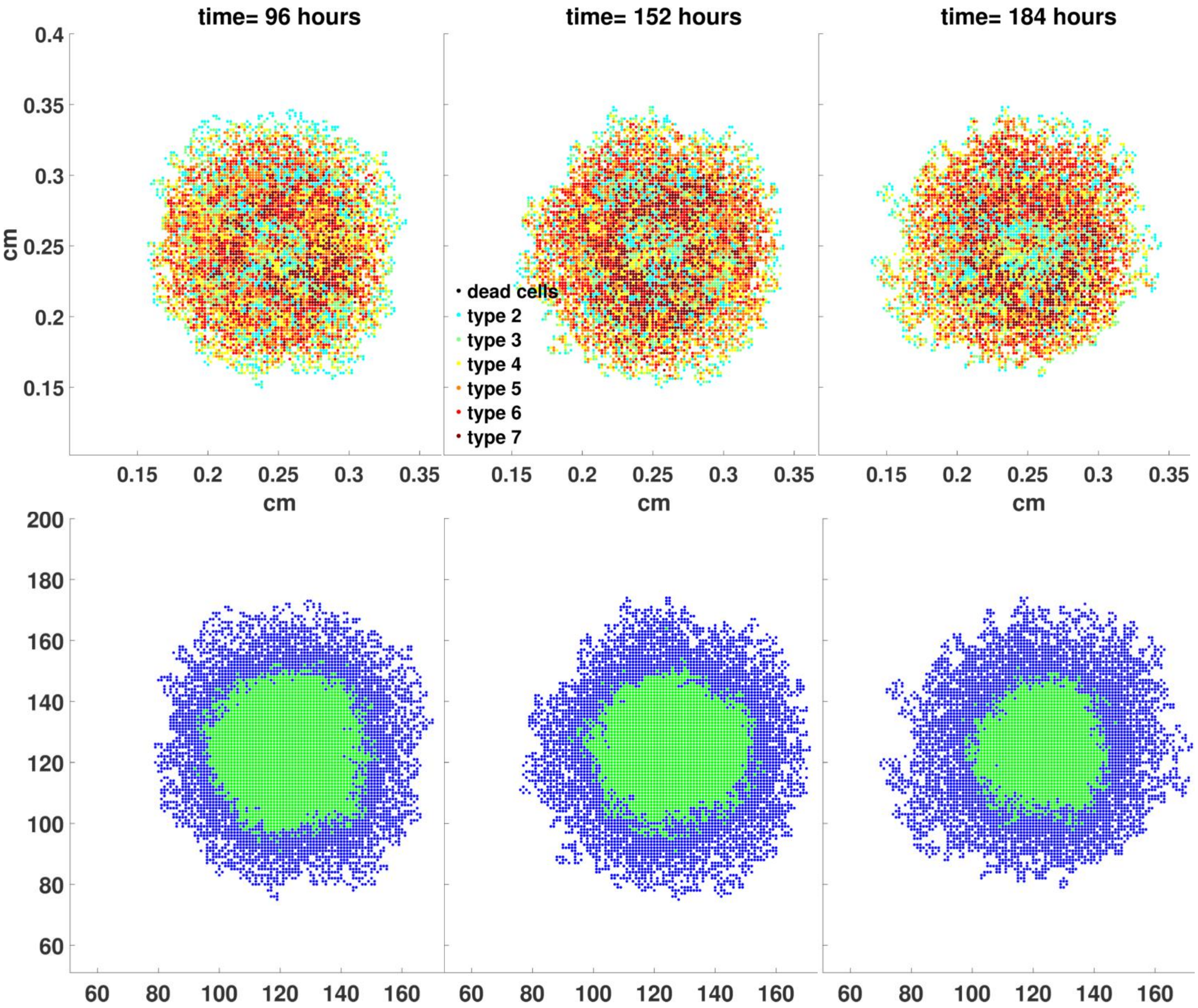

Figure Q: Sampled morphologies of the primary GB spheroids for the different proliferation rates examined. The snapshots were illustrate the morphology at the temporal point of first reaching 1 mm radius. The proliferation time was set at 15, 25 and 35 hours, respectively (left to right). The snapshots shown were taken at the iteration when cells reached 1 μm radius, at 96, 152 and 184 hours from left to right respectively.

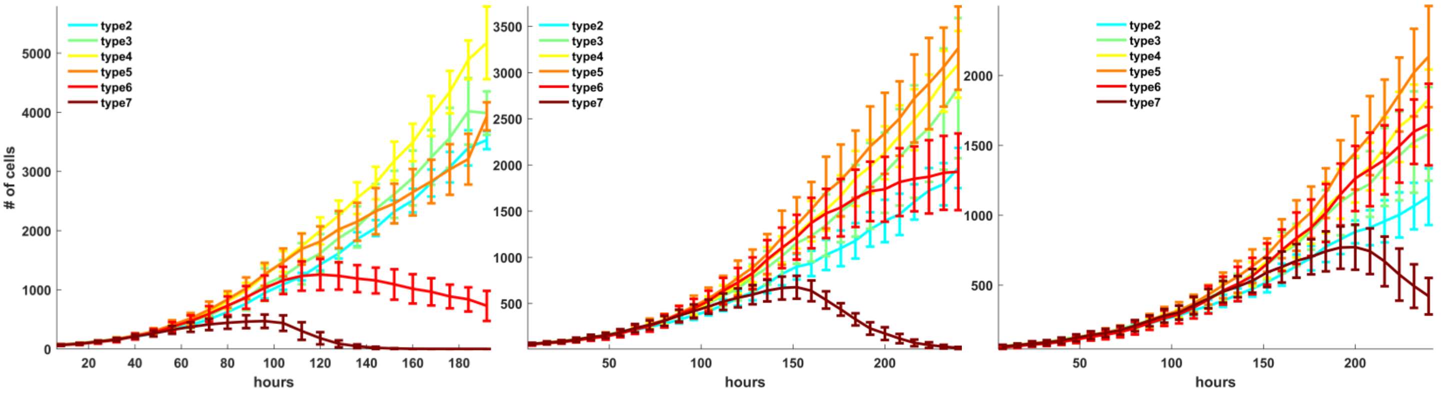

Figure R: Impact of the proliferation rate on the evolution of each phenotype for the primary GB cell line. Results from all the experiment are shown with proliferation time set at 15 (left), 25 (middle) and 35 hours (right). Note that as proliferation time decreases, the type 7 phenotype stops proliferating at earlier timepoint due to confinement.

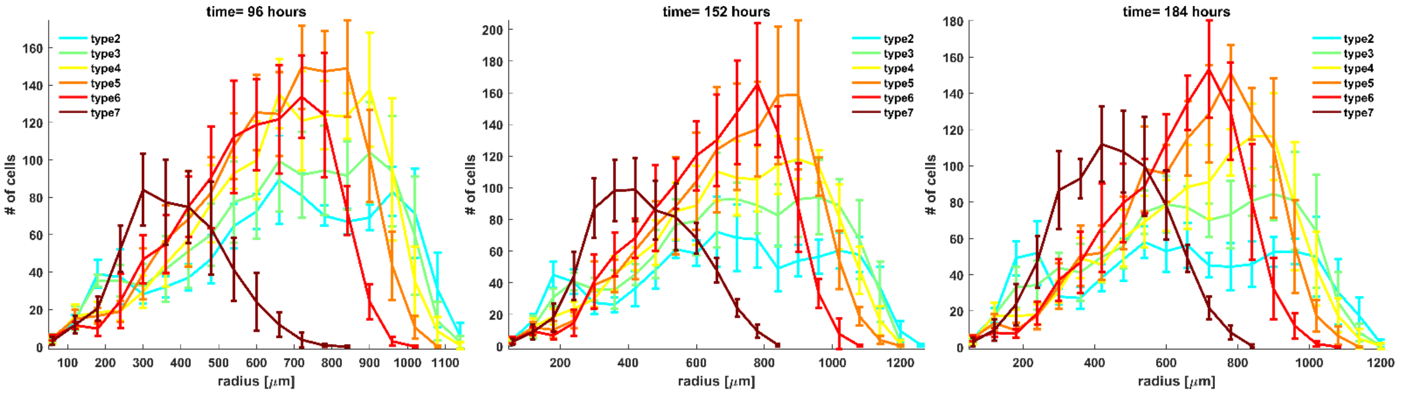

Figure S: Radial distribution of the phenotypes showing the impact of the proliferation rate for the primary GB cell line. Results from all experiments with proliferation rates set at 15 (left), 25 (middle) and 35 hours (right). The snapshots shown were taken at the iteration when cells reached 1 μm radius, at 96, 152 and 184 hours from left to right respectively.

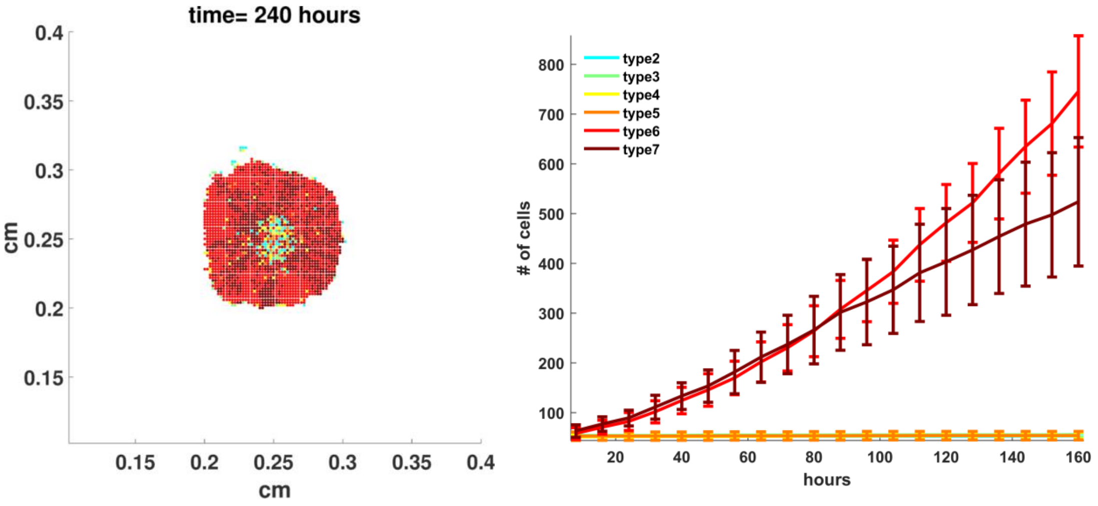

Figure T: Selective proliferation of the highly adhesive cells for the primary GB cell line. Snapshot of primary GB spheroid at 240 hours (Left) and the temporal evolution of each phenotype (Right). The dominance of the highly adhesive phenotypes (types 6 and 7), which are the only phenotypes that proliferate is evident.

Motility

Increasing the motility rate of the primary cells results in increase of both the invasive radius and the cell population as it allows more free space for cell growth and motility. Increase in motility rate considerably decreases the compactness of the spheroid and increases sparseness expressed with irregularities and roughness at the boundary. Interestingly, variations in the motility rates alter the relative frequency of each phenotype in the population as well as in terms of location.

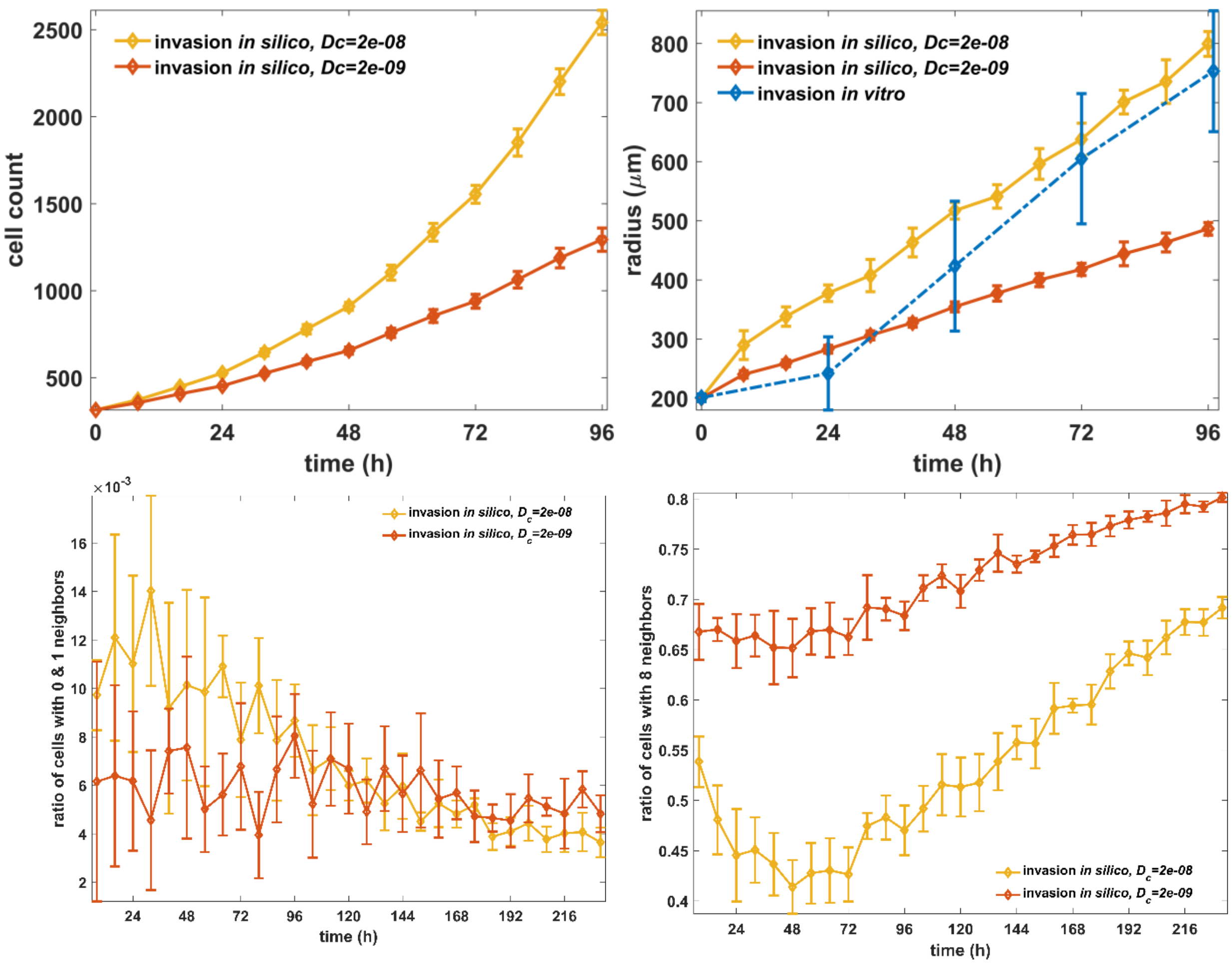

Figure U: Impact of the diffusion rate on the evolution of cell population and overall invasive area of the primary GB spheroids. Both the invasion area and the population increase when higher motility is enabled.

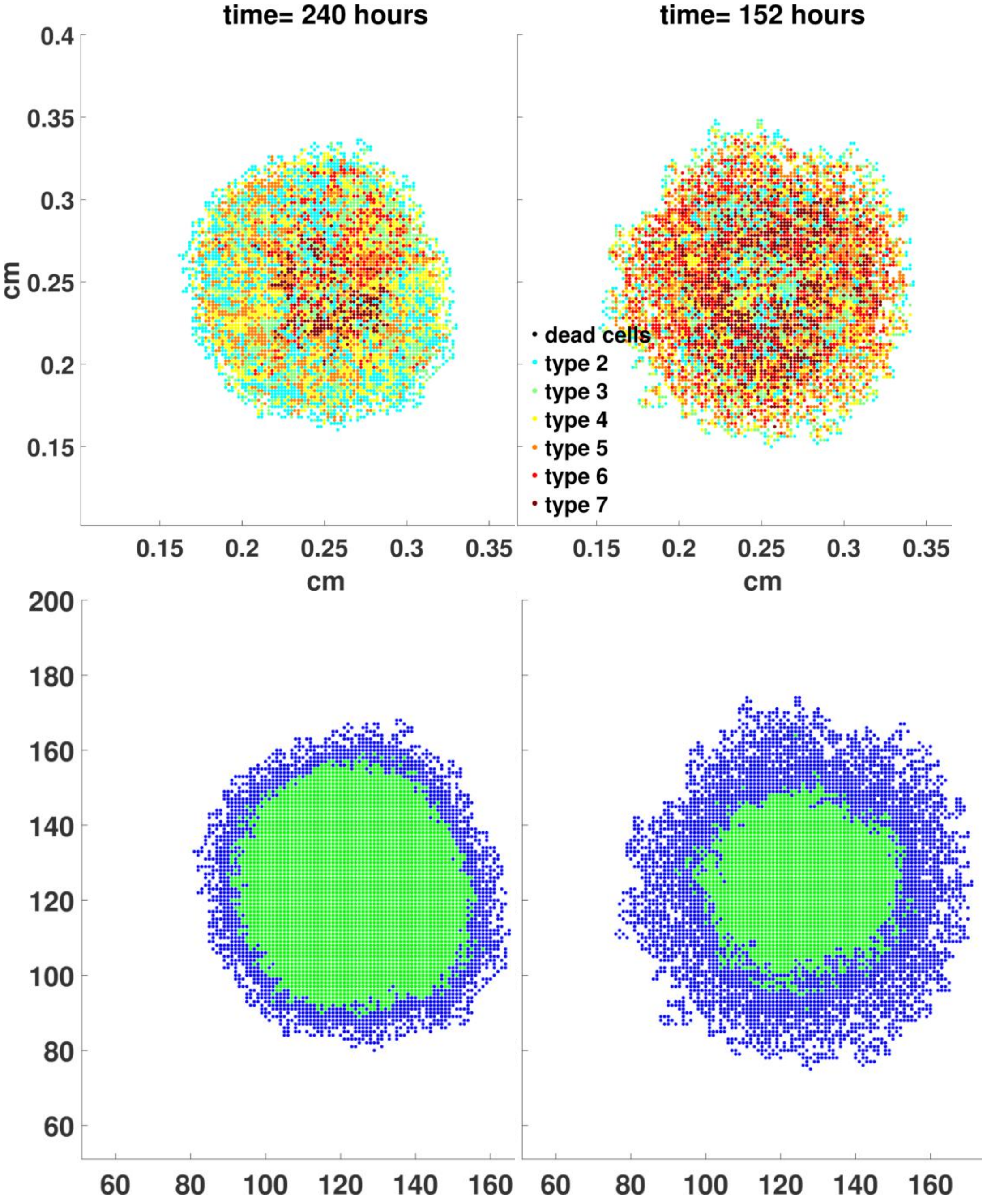

Figure V: Impact of the diffusion rate on the morphology of the primary GB spheroids. The diffusion rate was set at 2e-9 (left) and 2e-8 (right) cm^2/s, respectively. The snapshots illustrated are taken at 240 and 152 hours respectively. Decreased diffusion results in decreased tumor expansion and denser tumor center packed with quiescent cells. As the motility of type 7 phenotype is reduced due to its increased cell-to-cell adhesion interactions, type 7 phenotype is trapped in the tumor core unable to proliferate. In the higher diffusion rate scenario (right), the cells create gaps during movement, allowing for the type 7 cells to proliferate at the empty sites created. A necrotic core is also eventually formed due to the increased tumor size and compactness.

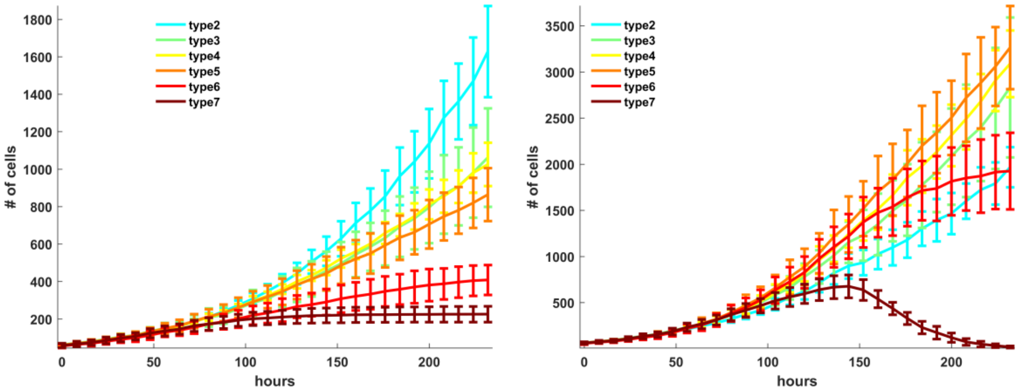

Figure W: Impact of the diffusion rate on the evolution of each phenotype of the primary GB cell line. Results from all experiment with diffusion rate set at 2e-9 (left) and 2e-8 (right) cm²/s, respectively. The proliferation of type 7 phenotype is inhibited as it is trapped in the tumor center. The relative dominance of type 2 phenotype is evident at low diffusion rate.

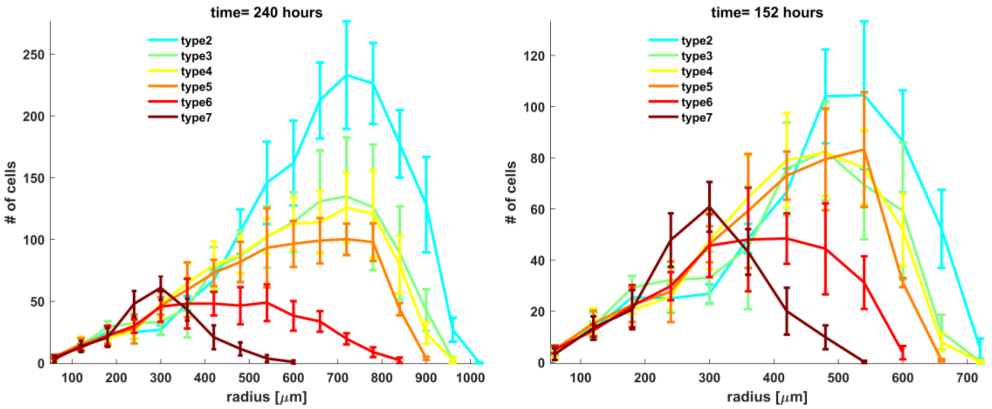

Figure X: Radial distribution of phenotypes showing the impact of the diffusion rate for the primary GB cell line. Results from all experiment with diffusion rate set at 2e-9 (left) and 2e-8 (right) cm²/s, respectively.

Phenotypic switch

An intrinsic state transition probability of tumor cells was introduced in this set of experiments that allows them to stochastically change phenotype during mitosis with probability equal to 0.5.

U87MG

U87MG cell population and invasive area are little affected by the mutation. The most noticeable difference is that after 56 hours the sparseness appears to stabilize for the experiment with phenotypic switching enabled.

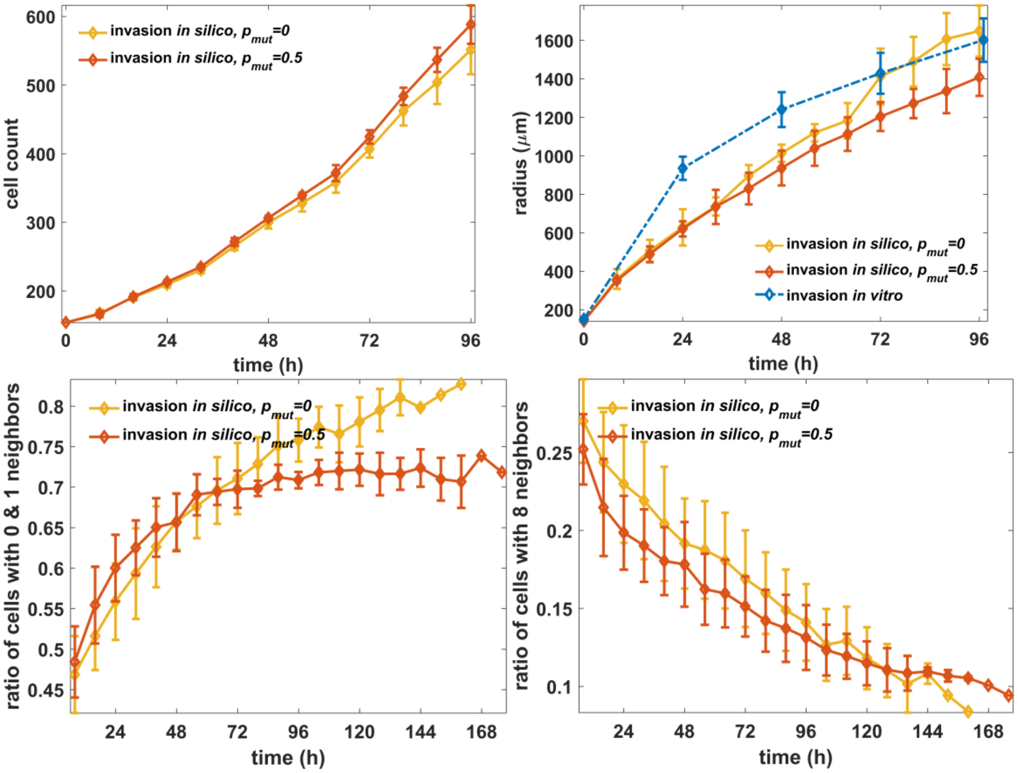

Figure Y: Impact of the phenotypic switch with respect to the adhesion preference on the evolution of cell population, the overall invasive area, the sparsity and the compactness of the U87MG spheroids. The case where no mutations are allowed in cell populations is also shown for comparison.

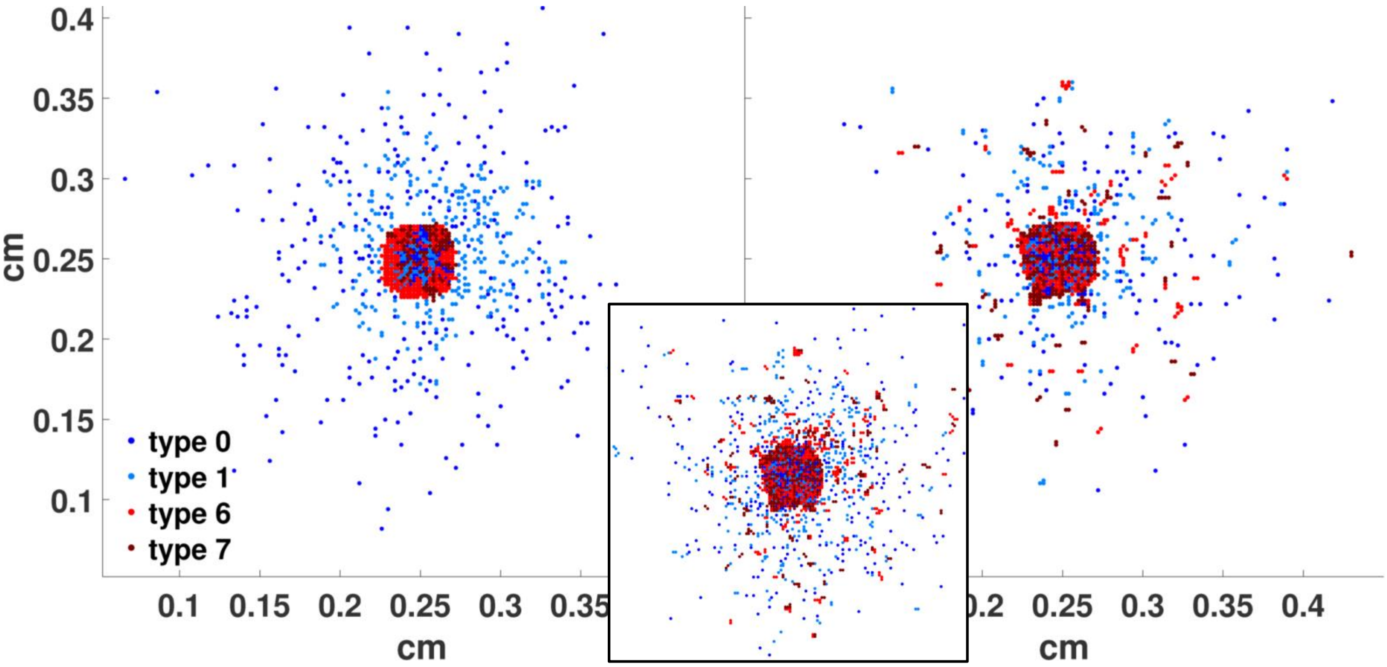

Figure Z: Examples of different morphologic conformations of the U87MG spheroids with and without allowing phenotypic switch. The two graphs are referred to 136 hours of simulation with mutation probability equal to 0 (left) and 0.5 (right), respectively. The graph in caption shows the final point of the mutation-enabled simulation at 176 hours. Notice the spots of cell aggregation around the core tumor, resembling the biological data, and emerged as an unsupervised computational result.

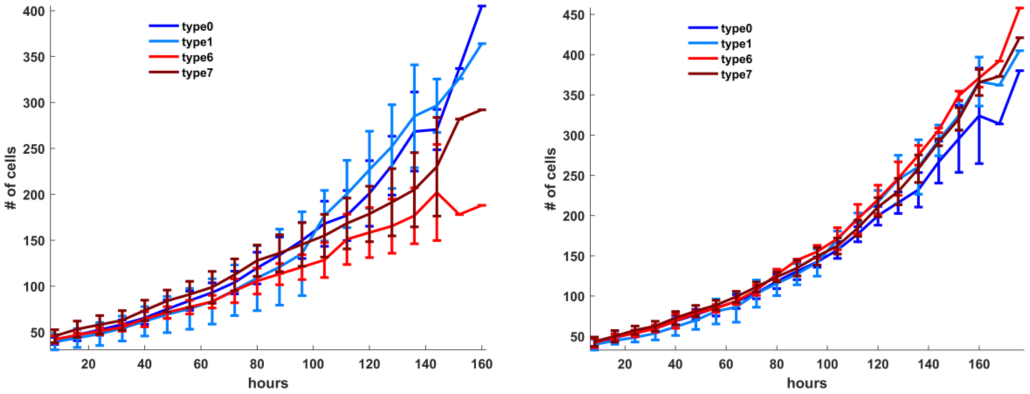

Figure AA: The evolution of the phenotypes with low (type 0 and type 1) and high (type 6 and type 7) adhesion values in the absence (left) and presence (right) of phenotypic switch of the U87MG spheroids.

Primary cells

Primary cells lose their emerging self-organization when phenotypic switching is enabled. Additionally the population and invasion area are higher without switching indicating that the resulting self-organization is more efficient.

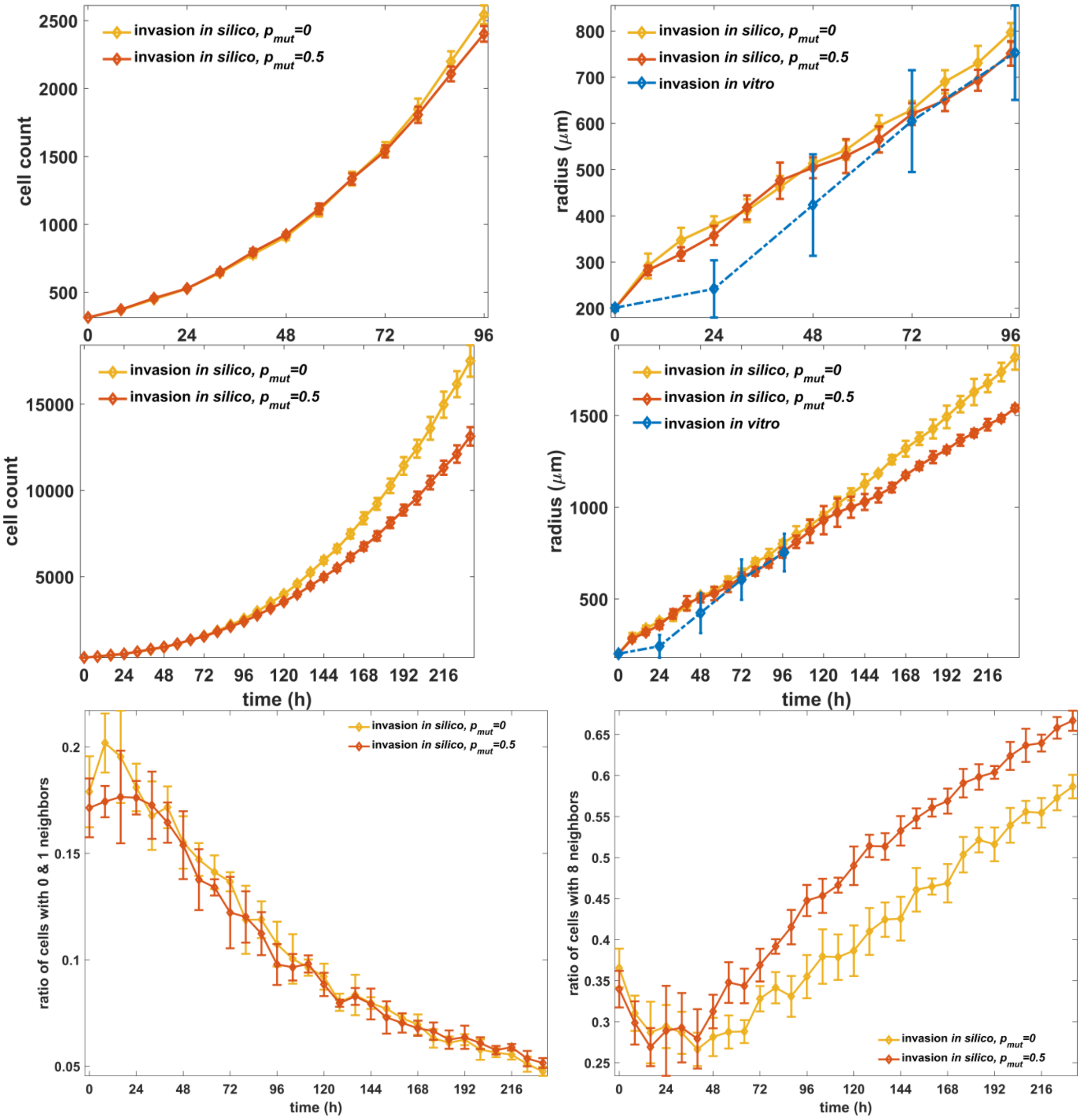

Figure BB: Impact of the phenotypic switch on the evolution of cell population and the overall invasive area, sparsity and compactness of the primary GB spheroids.

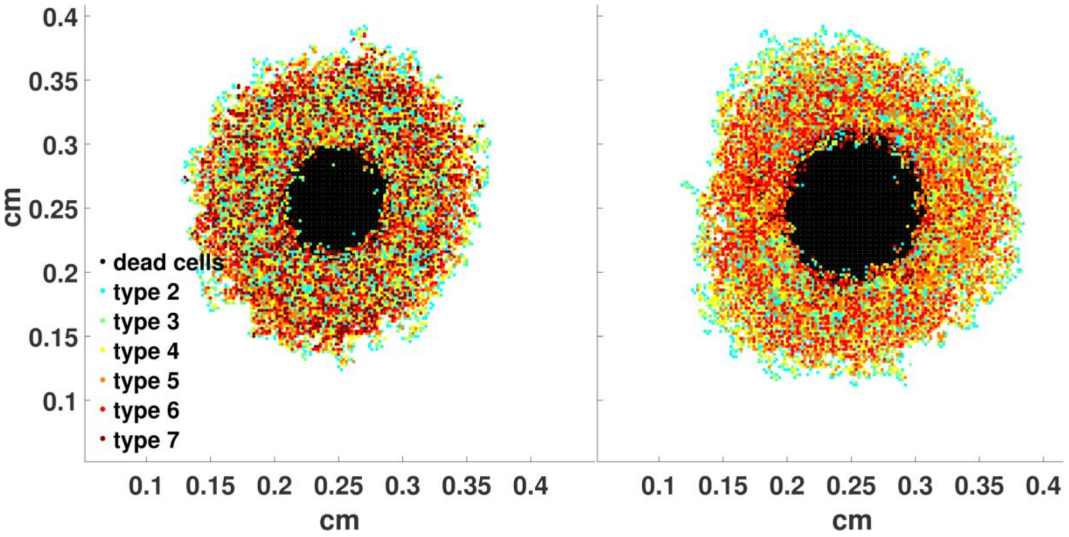

Figure CC: Examples of different morphologic conformations of the primary GB spheroids with and without the phenotypic switch. The two graphs are referred to 208 hours of simulation with mutation probability, p_{mut} equal to 0.5 (left) and 0 (right), respectively. When allowing phenotypic switch in the population, type 7 phenotype does not become extinct.

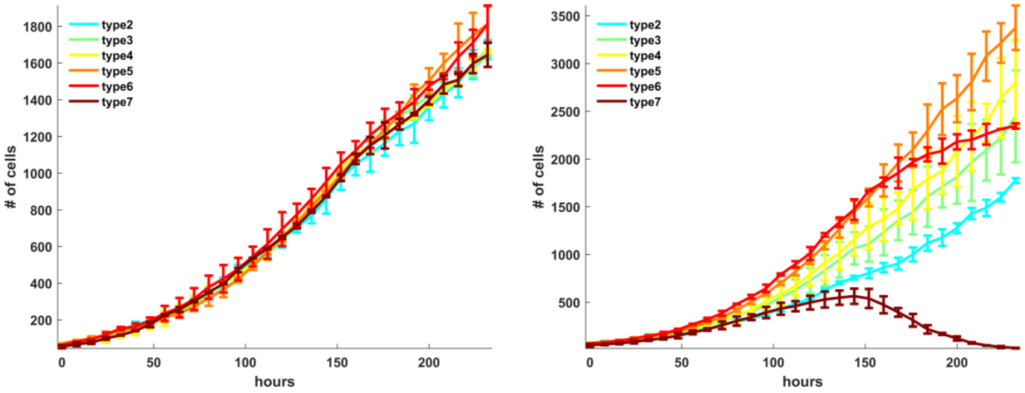

Figure DD: The temporal evolution of each phenotype of the primary GB cell line comprised of phenotypes with middle (type 2-5) and high (type 6 and type 7) adhesion values in the presence (left) and absence (right) of phenotypic switch.

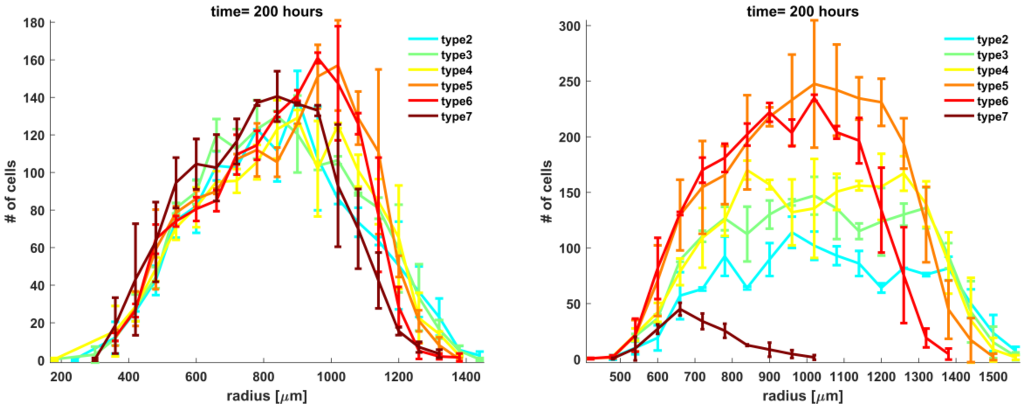

Figure EE: The radial distribution of phenotypes of the primary GB cell line comprised of phenotypes with middle (type 2-5) and high (type 6 and type 7) adhesion values in the presence (left) and absence (right) of phenotypic switch.

In vitro methods

Doubling time assay

The average doubling time intervals were estimated following a simple protocol in adherent cultures. A 24-well plate was seeded with 20000cells/ml of supplemented DMEM-F12 per cell type, incubated in standard lab conditions for approximately a week. Every 24 hours after plating, the content of one well per cell type was removed using trypsin-EDTA (Sigma-Aldrich, Germany) dissociation. The single cell solution suspension was permanently fixed using 4% formaldehyde and followingly, the cell concentration was measured using a hemocytometer. The procedure was repeated up to the point that 100% cell confluence was achieved. All the above measurements were repeated multiple times to better approximate the average doubling time of each cell line using exponential linear regression analysis (for more details, also see [1]).

In vitro images

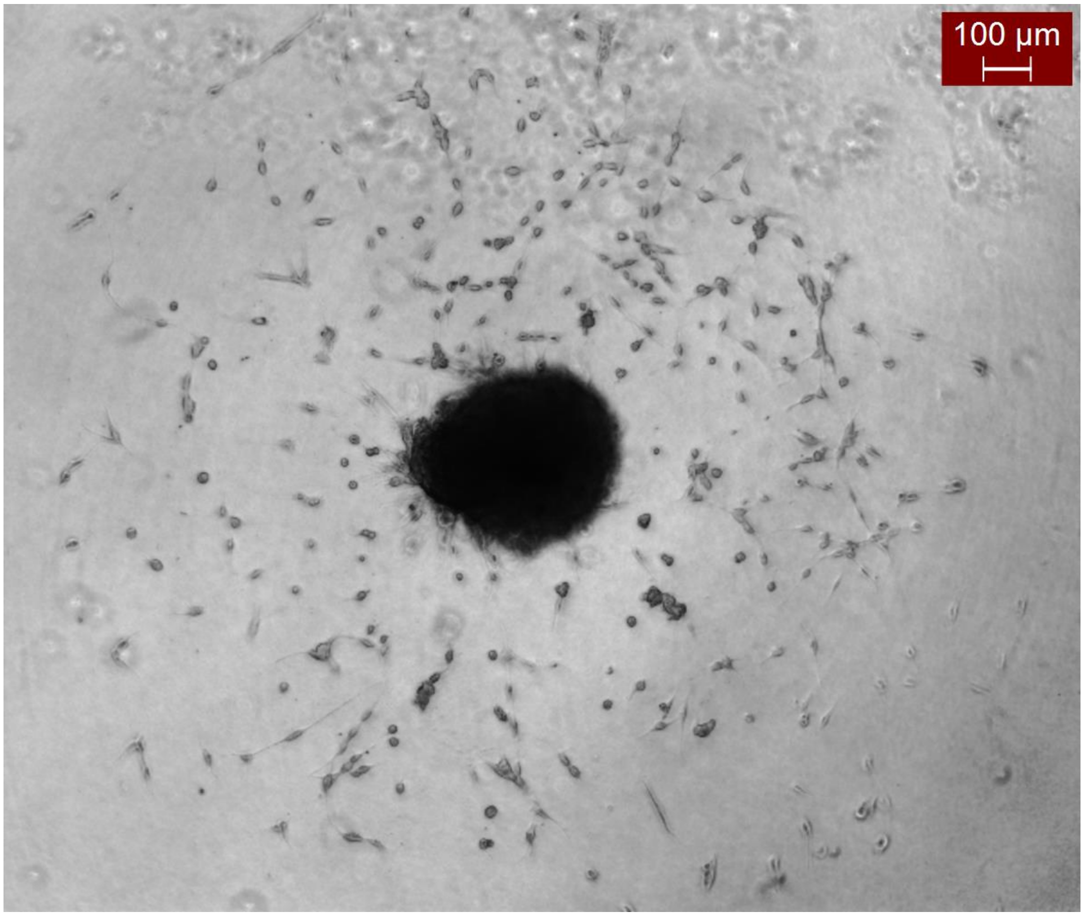

Figure FF. Invasion of U87MG at 96 hours.

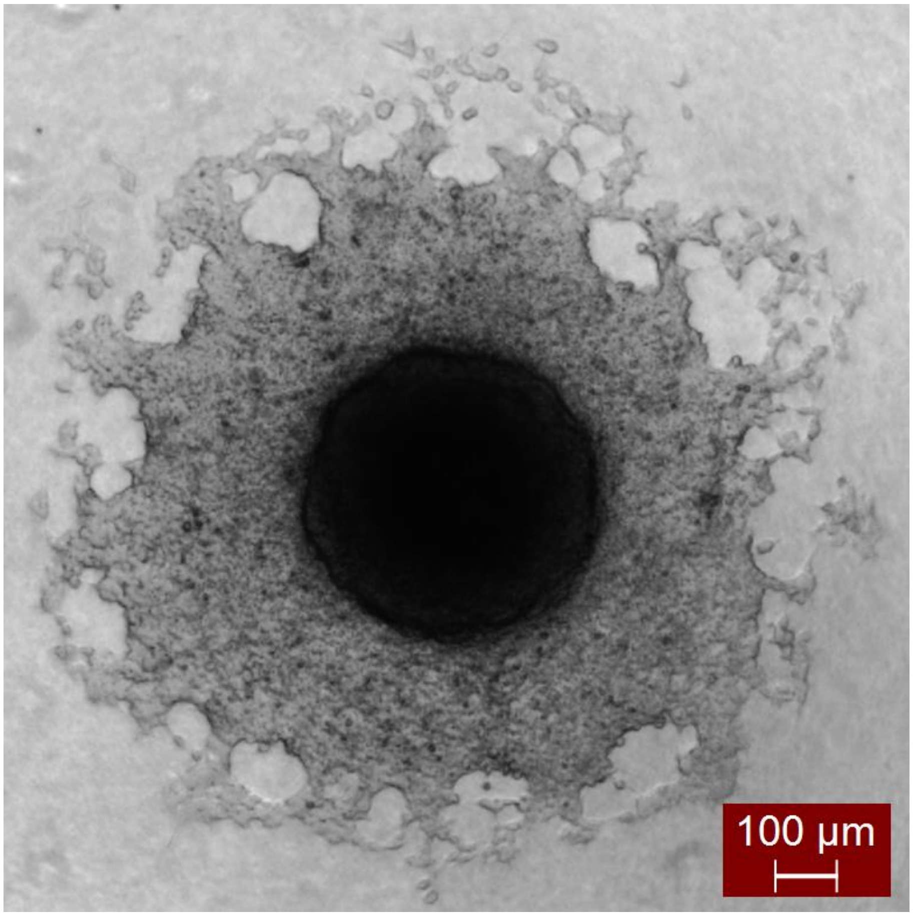

Figure GG: Invasion of primary GB spheroids at 96 hours.
